# Impaired Autophagic Flux in Skeletal Muscle of Plectin‐Related Epidermolysis Bullosa Simplex With Muscular Dystrophy

**DOI:** 10.1002/jcsm.70001

**Published:** 2025-07-10

**Authors:** Michaela M. Zrelski, Margret Eckhard, Petra Fichtinger, Sabrina Hösele, Andy Sombke, Leonid Mill, Monika Kustermann, Wolfgang M. Schmidt, Fiona Norwood, Ursula Schlötzer‐Schrehardt, Gerhard Wiche, Rolf Schröder, Lilli Winter

**Affiliations:** ^1^ Division of Cell and Developmental Biology, Center for Anatomy and Cell Biology Medical University of Vienna Vienna Austria; ^2^ MIRA Vision Microscopy GmbH Wangen Germany; ^3^ Neuromuscular Research Group, Division of Cell and Developmental Biology, Center for Anatomy and Cell Biology Medical University of Vienna Vienna Austria; ^4^ Department of Neurology, Ruskin Wing King's College Hospital London UK; ^5^ Department of Ophthalmology, University Hospital Erlangen Friedrich‐Alexander University Erlangen‐Nürnberg Erlangen Germany; ^6^ Department of Biochemistry and Cell Biology, max Perutz Laboratories University of Vienna Vienna Austria; ^7^ Institute of Neuropathology, University Hospital Erlangen Friedrich‐Alexander University Erlangen‐Nürnberg Erlangen Germany

**Keywords:** autophagy, desmin, epidermolysis bullosa simplex with muscular dystrophy, myofibrillar myopathy, plectin, skeletal muscle

## Abstract

**Background:**

Plectin, a multifunctional cytolinker and intermediate filament stabilizing protein, is essential for muscle fibre integrity and function. Mutations in the human plectin gene (*PLEC*) cause autosomal recessive epidermolysis bullosa simplex with muscular dystrophy (EBS‐MD). The disorganization and aggregation of desmin filaments in conjunction with degenerative changes of the myofibrillar apparatus are key features in the skeletal muscle pathology of EBS‐MD. We performed a comprehensive analysis addressing protein homeostasis in this rare protein aggregation disease by using human EBS‐MD tissue, plectin knock‐out mice and plectin‐deficient cells.

**Methods:**

Protein degradation pathways were analysed in muscles from EBS‐MD patients, muscle‐specific conditional plectin knockout (MCK‐Cre/cKO) mice, as well as in plectin‐deficient (*Plec* ^*−/−*^) myoblasts by electron and immunofluorescence microscopy. To obtain a comprehensive picture of autophagic processes, we evaluated the transcriptional regulation and expression levels of autophagic markers in plectin‐deficient muscles and myoblasts (RNA‐Seq, qRT‐PCR, immunoblotting). Autophagic turnover was dynamically assessed by measuring baseline autophagy as well as specific inhibition and activation in mCherry‐EGFP‐LC3B‐expressing *Plec* ^*+/+*^ and *Plec* ^*−/−*^ myoblasts, and by monitoring primary *Plec* ^*+/+*^ and *Plec* ^*−/−*^ myoblasts using organelle‐specific dyes. Wild‐type and MCK‐Cre/cKO mice were treated with chloroquine or metformin to assess the effects of autophagy inhibition and activation in vivo.

**Results:**

Our study identified the accumulation of degradative vacuoles as well as LC3‐ and SQSTM1‐positive patches in EBS‐MD patients, MCK‐Cre/cKO mouse muscles and *Plec* ^*−/−*^ myoblasts. The transcriptional regulation of ~30% of autophagy‐related genes was altered, and protein levels of downstream targets of the autophagosomal degradation machinery were elevated in MCK‐Cre/cKO muscle lysates (e.g., LAMP2, BAG3 and SQSTM1 to ~160, ~150 and ~140% of controls, respectively; *p* < 0.05). Autophagosome turnover was compromised in mCherry‐EGFP‐LC3B‐expressing *Plec* ^*−/−*^ myoblasts (~40% reduction in median red:green ratio, reduced puncta number, smaller puncta; *p* < 0.01). By labelling autophagic compartments with CYTO‐ID dye or lysosomes with LYSO‐ID, we found reduced signal intensities in primary *Plec* ^*−/−*^ cells (*p* < 0.001). Treatment with chloroquine led to drastic swelling of autophagic vacuoles in primary *Plec* ^*+/+*^ myoblasts, while the swelling in *Plec* ^*−/−*^ cells was moderate, establishing a defect in their autophagic clearance. Chloroquine treatment of MCK‐Cre/cKO mice corroborated that loss of plectin coincides with impaired autophagic clearance, while metformin amelioratively induced autophagic flux.

**Conclusions:**

Our work demonstrates that the characteristic protein aggregation pathology in EBS‐MD is linked to an impaired autophagic flux. The obtained results open a new perspective on the understanding of the protein aggregation pathology in plectin‐related disorders and provide a basis for further pharmacological intervention.

## 1 Introduction

Skeletal muscles are elaborately assembled machines possessing a precisely organized myofibrillar apparatus, designated for contraction and force generation. The principal components of the extrasarcomeric cytoskeleton are desmin intermediate filaments (IFs) forming a three‐dimensional scaffold around the myofibrillar Z‐disk and throughout the whole myofibre [1]. Plectin, an extraordinarily large (> 500 kDa) multimodular cytolinker protein, can be considered a central connector of the various cytoskeletal filament systems. It harbours a functional actin‐binding domain, binding sites for microtubule‐associated proteins and, most importantly, a binding domain for all types of IF proteins [2]. Distinct plectin isoforms in skeletal muscle bestow a central role in the interconnection but also in the targeting and anchorage of desmin IFs to sites of strategic importance, i.e., the Z‐disks (plectin 1d), costameres (plectin 1f), mitochondria (plectin 1b) and the nuclear/ER membrane system (plectin 1) [3, 4, 5, 6]. Thus, plectin orchestrates the structural and functional organization of filamentous cytoskeletal networks, IFs in particular, and thereby substantially contributes to the fundamental biomechanical properties of stress‐bearing tissues such as muscle.

As a consequence, the ablation or expression of mutant plectin in human and mouse leads to a faulty organization of the desmin IF system in striated muscle, thereby inflicting a reduced mechanical stress tolerance and a progressive myopathic process. Accordingly, pathogenic sequence alterations of the human plectin gene (*PLEC*) cause a multitude of clinical entities subsumed under the term ‘plectinopathies’. Epidermolysis bullosa simplex with muscular dystrophy (EBS‐MD, MIM #226670), a rare autosomal‐recessive disease with congenital skin blistering and late‐onset progressive muscle weakness, is the most prevalent plectinopathy [7, 8]. Due to its universal expression, diseases caused by *PLEC* mutations frequently display multisystemic manifestations including more and more additional symptoms [7]. The morphological hallmark of the skeletal muscle pathology in most plectinopathies is the presence of desmin‐positive protein aggregates, degenerative changes of the myofibrillar apparatus and mitochondrial abnormalities [5, 7, 9, 10, 11].

While loss of IF network function in conjunction with increased mechanical vulnerability and protein aggregation unambiguously promotes the progressive muscle damage in EBS‐MD, imbalanced protein homeostasis is highly anticipated to play a role in the pathogenesis of the disease. To this end, we examined protein quality control mechanisms, such as autophagy, in skeletal muscle specimens from EBS‐MD patients, muscle‐specific conditional plectin knockout mice (MCK‐Cre/cKO) and plectin‐deficient myoblasts.

## 2 Materials and Methods

### 2.1 Human Skeletal Muscle Biopsy Material

Tissue samples of previously reported EBS‐MD patients [9, 11, 12] were obtained from the Institute of Neuropathology, University Hospital Erlangen, and the Department of Neurology, King's College Hospital London, and were used for immunofluorescence and electron microscopy as described in the Supporting Information. Human plectin reference sequence: NM_000445.5; *PLEC* mutations are listed in the Supporting Information. The study was approved by the local ethics committees of participating institutions and conducted according to the principles of the Declaration of Helsinki (2013). Written informed consent was obtained from all participants.

### 2.2 Animals

Wild‐type mice homozygous for the floxed plectin allele (*Plec* ^*fl/fl*^) [13] or MCK‐Cre/cKO [5], both in a C57BL/6 background, were housed under specific and pathogen‐free conditions in a standard environment with free access to water and food and handled in accordance with the Austrian Federal Government laws and regulations. Animals were sacrificed through cervical dislocation, and muscles were subsequently processed for analyses as described in the Supporting Information. To determine in vivo the autophagic flux, 20‐week‐old wild‐type and MCK‐Cre/cKO animals were injected intraperitoneally with 0.9% saline (control; Sigma‐Aldrich, S8776) or with CQ (10 mg/kg in 0.9% saline; Sigma‐Adrich, C6628) 4 h ante mortem [14]. For metformin treatment, 11‐week‐old animals were switched to water supplemented with 0.2 g/100 mL sucrose for 14 days prior to the start of the experiment. Water was exchanged every 7 days, and the water consumption was monitored by weighing water bottles. The metformin concentration attributing to 500 mg/kg/day was calculated from the prior water consumption and the average body weight of the animals within one cage, supplemented at Day 0, and then exchanged and adapted (for the average animal weight and the water consumption) at weekly intervals for a total duration of 30 days. All experiments involving animals were performed according to Austrian Federal Government laws and regulations and were approved by the Austrian Federal Ministry of Education, Science and Research (BMBWF‐66.009/0226‐V/3b/2019 and 2022‐0.660.178).

### 2.3 Cell Culture

Immortalized skeletal myoblasts were derived from plectin‐expressing (*Plec* ^*+/+*^) or plectin‐deficient (*Plec* ^*−/−*^) littermates, both crossed into a p53‐deficient (*p53* ^*−/−*^) background, as described [10]. To generate myoblast cell lines stably expressing pBabe puro mCherry‐EGFP‐LC3B (from J. Debnath [Addgene, 22418] [15]), retroviral supernatants were generated by transfecting phoenix eco cells as previously described [16], with the modification that viral particles were released into F‐10‐based growth medium. Primary myoblasts were isolated from de‐skinned front and hind limbs of neonatal *Plec* ^*+/+*^ and *Plec* ^*−/−*^ mice as described [10]. Primary human dermal fibroblasts from EBS‐MD patients [17, 18] were obtained from the Department of Dermatology, Medical Center University of Freiburg and cultivated as described [19]. *PLEC* mutations are listed in the Supporting Information. The study was approved by the ethics committee of the University of Freiburg (ethics number 293‐14) and conducted according to the principles of the Declaration of Helsinki (2013). Written informed consent was obtained from all participants. Myoblast and fibroblast cell cultures were used for analyses as described in the Supporting Information.

## 3 Results

### 3.1 Increased Autophagic Build‐Up in Skeletal Muscles From EBS‐MD Patients

**FIGURE 1:**
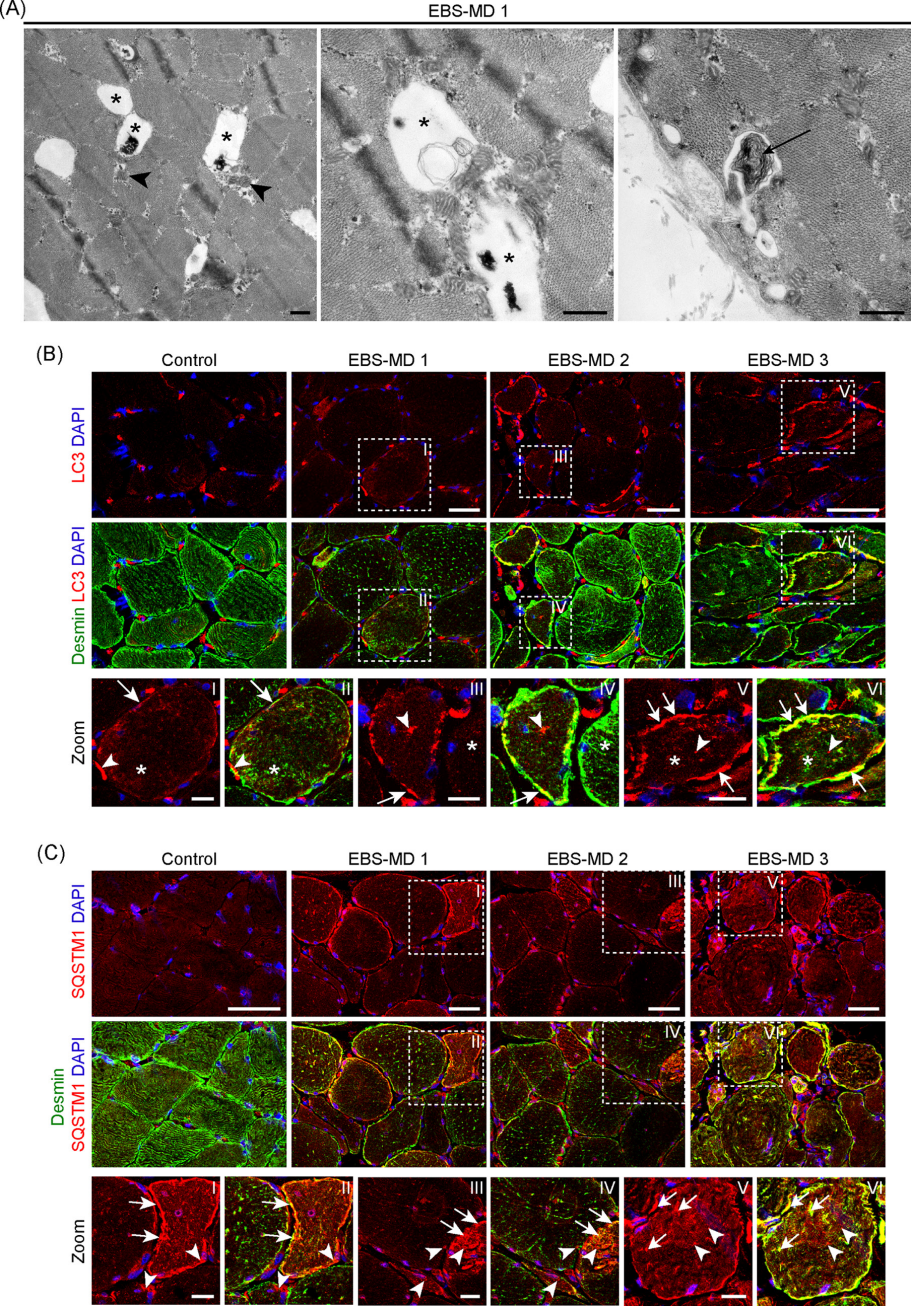
Identification of degradative vacuoles and accumulation of autophagy marker proteins in EBS‐MD patient muscle. (A) Representative electron micrographs of muscle sections derived from EBS‐MD patient 1 (EBS‐MD 1) displaying various degradative vacuoles (asterisks), partially filled with cytoplasmic material and/or membrane remnants and often associated with damaged mitochondria (arrowheads), as well as the occurrence of myelinated bodies (arrow). Scale bars: 500 nm. (B) Double immunostaining of frozen muscle sections derived from a healthy control (Control) and EBS‐MD patients (EBS‐MD 1, EBS‐MD 2, EBS‐MD 3) using antibodies to LC3 (red) and desmin (green). Nuclei were visualized using DAPI. Boxed areas indicate magnifications of individual muscle fibres (as denoted by the boxes I–VI). Note the accumulation of LC3‐positive patches within EBS‐MD patient myofibres, either colocalizing with desmin aggregates (arrows) or without desmin association (arrowheads). Also, note the occurrence of desmin‐positive protein aggregates not associating with LC3 protein signals (asterisks). Scale bars: 50 μm; magnifications I–VI 20 μm. (C) Double immunostaining of frozen muscle sections derived from a healthy control and EBS‐MD patients using antibodies to SQSTM1 (red) and desmin (green). Nuclei were visualized using DAPI. Boxed areas indicate magnifications of individual muscle fibres (as denoted by the boxes I–VI). Note the occurrence of individual EBS‐MD myofibres displaying massive SQSTM1‐positive areas, while others harbour small SQSTM1‐positive aggregates, either colocalizing with desmin aggregates (arrows) or without desmin association (arrowheads). Scale bars: 50 μm; magnifications I–VI 20 μm.

Ultrastructural evaluation of a muscle biopsy from an EBS‐MD patient [11] showed numerous autophagic vacuoles in the sarcoplasma of myofibres, either filled with cargo remnants appearing as electron‐dense deposits or membrane fragments, and frequently located in close proximity to damaged mitochondria (Figure 1A). In addition, subsarcolemmal vacuoles containing myelinated bodies were present. Immunostaining of cryopreserved muscle sections from this as well as from two additional EBS‐MD patients [9, 11, 12] showed a marked disruption of the desmin IF networks in conjunction with desmin‐positive protein aggregates. Microscopic analysis of LC3, marking autophagosomal structures, revealed a diffuse sarcoplasmic labelling in a subset of EBS‐MD muscle fibres as well as a perinuclear labelling, which was also observed in control samples (Figure 1B). In addition, focal deposits of LC3 were frequently noted in EBS‐MD muscles, while no LC3 accumulation was seen in control samples and diffuse sarcoplasmic staining was consistently absent. Staining intensities of SQSTM1, a protein that connects polyubiquitinated proteins with LC3, were elevated in all EBS‐MD patient muscles, with some fibres displaying massive SQSTM1‐labelled subsarcolemmal as well as focal dense sarcoplasmic staining patterns (Figure 1C). While a significant proportion of the LC3‐positive patches or SQSTM1‐positive deposits clearly co‐localized with desmin‐positive aggregates (Figure 1B,C, magnifications I–VI, arrows), the majority appeared independent of the collapsed IF network (Figure 1B,C, magnifications I–VI, arrowheads). Taken together, intense LC3 and SQSTM1 immunoreactivity and ultrastructural evidence of abundant autophagic vacuoles suggested increased autophagic build‐up in skeletal muscles of EBS‐MD patients.

### 3.2 Plectin‐Deficient Mouse Muscles Mirror the Autophagy‐Related Pathology in Human EBS‐MD Muscles

**FIGURE 2:**
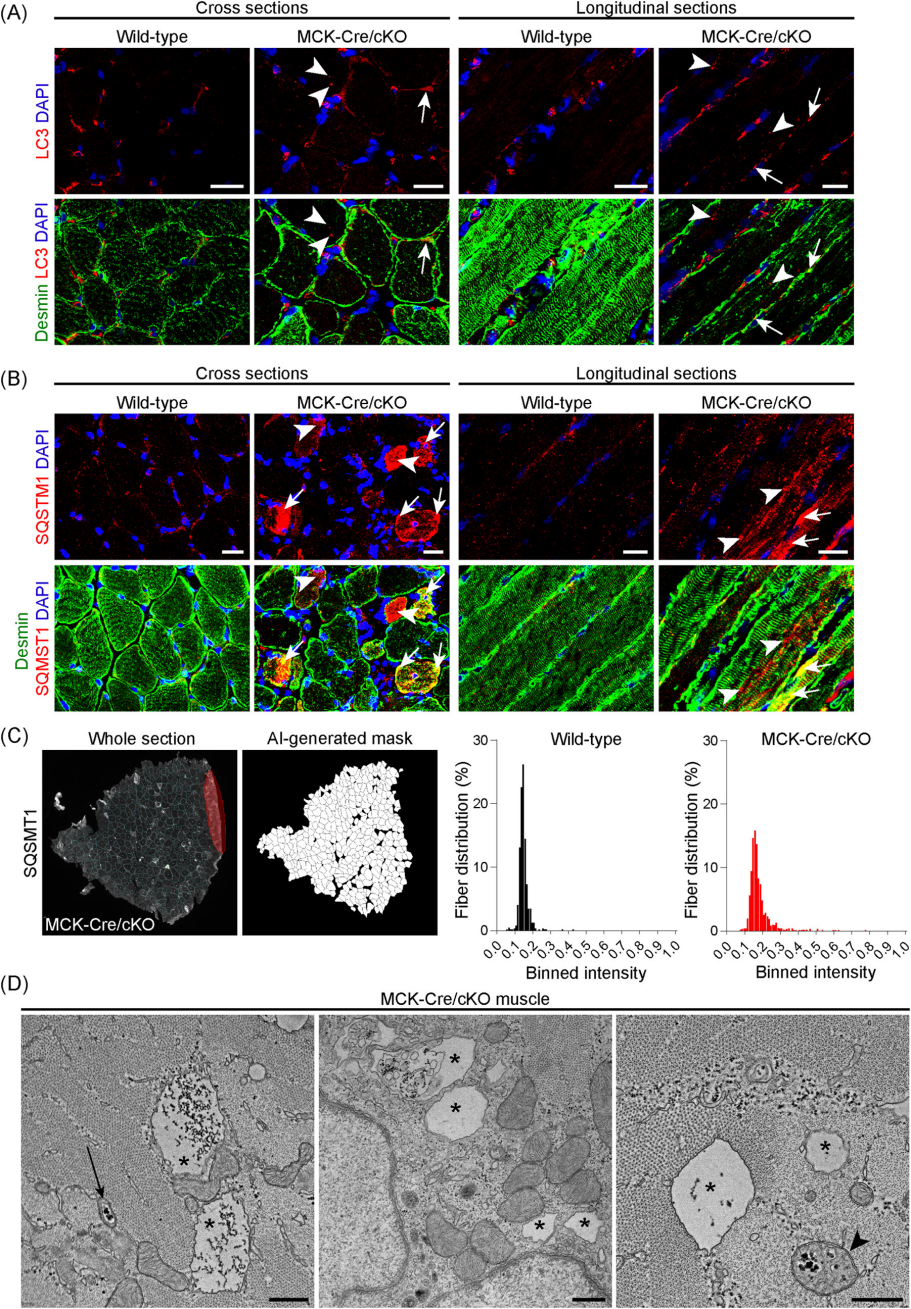
Accumulation of autophagy marker proteins and ultrastructural visualization of degradative vacuoles in plectin‐deficient mouse muscle. (A) Double immunostaining of cross and longitudinal soleus muscle sections from wild‐type and plectin‐deficient (MCK‐Cre/cKO) mice using antibodies to LC3 (red) and desmin (green). Nuclei were visualized with DAPI. Note the accumulation of LC3‐positive patches in plectin‐deficient muscles, either colocalizing with desmin aggregates (arrows) or without desmin association (arrowheads). Scale bars: 20 μm. (B) Double immunostaining of cross and longitudinal soleus muscle sections from wild‐type and MCK‐Cre/cKO animals using antibodies to SQSTM1 (red) and desmin (green). Nuclei were visualized with DAPI. Note the occurrence of individual plectin‐deficient myofibres displaying massive SQSTM1‐positive areas, while others harbour small SQSTM1‐positive aggregates, either colocalizing with desmin aggregates (arrows) or without desmin association (arrowheads). Scale bars: 20 μm. (C) Artificial intelligence (AI)‐based evaluation of SQSTM1 signal intensities within individual myofibres using MIRA Vision. Whole soleus muscle sections were scanned, myofibres automatically identified in an AI‐generated mask, and signal intensities obtained and binned for each genotype (bin size = 0.1). Histograms represent the frequency distribution of binned intensities obtained from two animals each (wild‐type, *n* = 673 fibres; MCK‐Cre/cKO, *n* = 1128 fibres). Note the expanded distribution of the histogram and a shift towards the right (i.e., higher intensities) for plectin‐deficient muscles. (D) Representative electron micrographs of soleus muscle cross sections obtained from 40‐week‐old MCK‐Cre/cKO mice. Note the occurrence of various degradative vacuoles, including vacuoles with their cargo engulfed in a double‐membrane (arrow), and pathologically enlarged vacuoles that are partially filled with glycogen and/or membrane remnants (asterisks). In addition, pathologically altered mitochondria with inclusions can be observed (arrowhead). Scale bars: 500 nm.

In cross and longitudinal soleus muscle sections from 13‐week‐old MCK‐Cre/cKO mice, an established model for the human EBS‐MD muscle pathology [5], a focal accumulation of LC3‐positive patches was noted (Figure 2A). While some LC3‐stained deposits colocalized with desmin‐labelled aggregates, the majority appeared independent of the collapsed desmin IF network. Perinuclear LC3 stains were detected in wild‐type and diseased muscle samples. Likewise, microscopic analyses of SQSTM1 revealed plectin‐deficient muscle fibres with markedly accumulated protein signals, only partially co‐localizing with desmin aggregates (Figure 2B). The LC3 and SQSTM1 patterns in plectin‐deficient muscles thus strongly mirror the results obtained in EBS‐MD muscle. We then quantified the SQSTM1 signal intensities in whole muscle sections with MIRA Vision (https://www.mira.vision/), an artificial intelligence (AI)‐based system, and analysed the frequency distribution of SQSTM1‐positive immunosignals from individual myofibres by binning and plotting the relative signal intensities as histograms (Figure 2C). Compared with wild‐type, the MCK‐Cre/cKO histogram displayed an expanded distribution and a clear shift towards higher binned intensities, highlighting a higher percentage of intensely‐labelled myofibres. Notably, a similar distribution was obtained when the muscle biopsy from EBS‐MD patient 1 was subjected to AI‐based evaluation (Figure S1A). In addition, transmission electron microscopy analyses of soleus muscles from aged MCK‐Cre/cKO mice showed an increased autophagic build‐up (Figure 2D; lower magnification electron micrographs of MCK‐Cre/cKO muscles as well as control samples are shown in Figure S1B,C). Here, autophagic vacuoles were partially filled with glycogen and/or membrane remnants, and their cargo was engulfed by a double‐membrane. Again, morphologically altered mitochondria, sometimes containing inclusions, were frequently observed in close vicinity to autophagic vacuoles.

### 3.3 Alterations in the Transcriptional Regulation of Autophagy in MCK‐Cre/cKO Muscles

**FIGURE 3:**
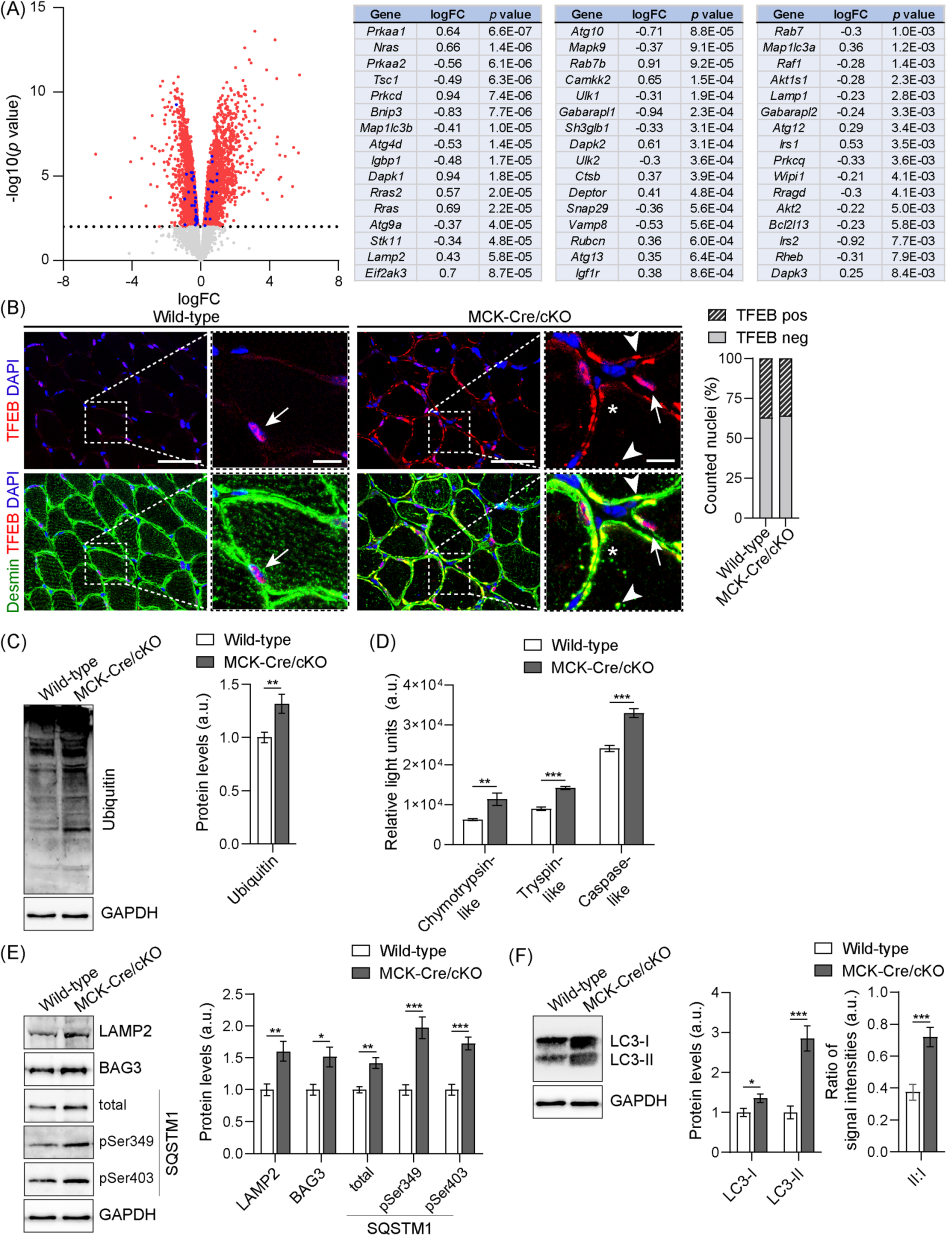
Increased protein levels of autophagic substrates in MCK‐Cre/cKO muscles. (A) RNA‐Seq analysis of mouse soleus muscle. Volcano plot illustrates differentially expressed genes in MCK‐Cre/cKO compared with wild‐type samples: significantly upregulated and downregulated genes are highlighted in red; the dotted line represents the cut‐off with *p =* 0.01. Genes from the Kyoto Encyclopedia of Genes and Genomes (KEGG) pathway ‘mmu04140 Autophagy ‐ animal’ are highlighted in blue and listed on the right. logFC, log fold change; *n* = 5 animals per genotype, sequenced individually. (B) Double immunostaining of soleus muscle sections from wild‐type and MCK‐Cre/cKO mice using antibodies to TFEB (red) and desmin (green). Nuclei were visualized with DAPI. Panels on the right are magnifications of boxed areas indicated in the panels on the left. Note that while a nuclear localization of TFEB was preserved in both genotypes (arrows), concentrated TFEB‐positive subsarcolemmal and intermyofibrillar areas occurred only in MCK‐Cre/cKO fibres. Also note that some desmin‐positive aggregates colocalized with TFEB‐positive signals (arrowheads), while others did not. Scale bars: 50 μm, magnifications 10 μm. Quantification of nuclei with or without TFEB localization (wild‐type, *n* = 1082 nuclei; MCK‐Cre/cKO, *n* = 1466 nuclei; two animals each). (C) Immunoblotting of wild‐type and MCK‐Cre/cKO muscle lysates using antibodies to ubiquitin and GAPDH. Signal intensities of immunoblots were densitometrically measured and normalized to the total protein content as analysed by Coomassie staining (not shown). Mean ± SEM; *n* = 8. (D) Chymotrypsin‐, trypsin‐, and caspase‐like proteasomal activities were measured in wild‐type and MCK‐Cre/cKO muscle lysates derived from 13‐week‐old mice. Mean ± SEM; samples were measured as triplicates, *n* = 3 animals each. (E) Immunoblotting of wild‐type and MCK‐Cre/cKO muscle lysates using antibodies to LAMP2, BAG3, total and two phosphorylated forms of SQSTM1 and GAPDH. Signal intensities of protein bands were densitometrically measured and normalized to the total protein content as analysed by Coomassie staining (not shown). Mean ± SEM; *n* = 7–8. (F) Immunoblotting of wild‐type and MCK‐Cre/cKO muscle lysates using antibodies to LC3 and GAPDH. Signal intensities of upper (nonlipidated, LC3‐I) and lower (lipidated, LC3‐II) protein bands were densitometrically measured and normalized to the total protein content (as analysed by Coomassie staining, not shown). From these values, the LC3‐II to LC3‐I ratios were calculated. Mean ± SEM; *n* = 10. For C–F: *p < 0.05, **p < 0.01, ***p < 0.001 (two‐tailed, unpaired *t* test with Welch's correction); ns, not significant.

To explore if autophagy‐related genes (ATGs) or pathways were altered in plectinopathies, we employed RNA‐sequencing (RNA‐seq)–based transcriptomic analyses of wild‐type and MCK‐Cre/cKO muscles. Differential expression analysis using *edgeR* revealed 2169 genes up‐ and 2215 genes downregulated in MCK‐Cre/cKO soleus muscle samples (Figure 3A). Gene set enrichment analysis (GSEA) of hallmark gene sets, representing well‐defined biological states from the Mouse Molecular Signatures Database, identified multiple pathways significantly altered between wild‐type and MCK‐Cre/cKO mice (Figure S2A). Likewise, a complementary analysis using gene sets from the Gene Ontology (GO) Biological Process (BP) subcollection revealed more than 1500 pathways significantly altered, including the three autophagy‐related GOBP pathways ‘autophagy of mitochondrion’, ‘regulation of autophagic cell death’ and ‘regulation of autophagy of mitochondrion in response to mitochondrial depolarization’. Supervised GSEA focusing on autophagy revealed that 34 of 107 genes (31.8%) within the ‘macroautophagy’ pathway, 37 of 120 genes (30.8%) within the ‘autophagy’ pathway, and 12 of 31 genes (38.7%) within the ‘autophagy of mitochondrion’ pathway were differentially expressed (Figure S2B). Functional annotation by using the Kyoto Encyclopedia of Genes and Genomes (KEGG) “mmu04140 Autophagy – animal” pathway analysis identified one third (*n* = 47) out of 142 genes differentially expressed (enlisted in Figure 3A). Moreover, the transcription factor EB (TFEB), a key molecule regulating the autophagy‐lysosomal pathway at the transcriptional level [20], accumulated in subsarcolemmal regions of MCK‐Cre/cKO muscles; in addition small TFEB‐positive deposits within myofibres were found, both partially colocalizing with desmin‐positive aggregates (Figure 3B). However, the number of TFEB‐positive nuclei was in the same range in both wild‐type and MCK‐Cre/cKO muscles. Even though the phosphorylated, nonactive confirmation of TFEB (pTFEB) appeared increased to ~115% in plectin‐deficient muscles, the protein levels of unphosphorylated, active TFEB were similar in both genotypes (Figure S3A). Together, these data imply that the skeletal muscle pathology in MCK‐Cre/cKO muscles is associated with marked transcriptional changes in autophagy‐relevant genes.

### 3.4 Accumulation of Autophagy Marker Proteins and Increased Proteasomal Function in MCK‐Cre/cKO Muscles

When the expression levels of PQC‐related proteins required for the induction of autophagy or the build‐up of the phagophore, such as mTOR, ULK1, ATG7, Beclin‐1, ATG5 and ATG3 [21], were quantitatively assessed in lower leg muscle (gastrocnemius and soleus) lysates, all evaluated proteins revealed no obvious differences between the two genotypes, indicating unaltered autophagy initiation (Figure S3B). In contrast, the amounts of ubiquitinated proteins were ~1.3‐fold increased in MCK‐Cre/cKO compared with wild‐type muscle lysates (Figure 3C). In addition, while mRNA levels of proteasomal components were largely unaltered (Figure S3C), chymotrypsin‐, trypsin‐ and caspase‐like proteasomal activities were significantly elevated to ~180%, ~155% and ~140% in MCK‐Cre/cKO compared with wild‐type muscles, respectively (Figure 3D). When normalized to the total levels of proteasomes (increased to ~130% in MCK‐Cre/cKO; Figure S3D), the proteasomal activities were basically unchanged in MCK‐Cre/cKO compared with wild‐type muscles (Figure S3E), indicating that plectin deficiency leads to increased levels of proteasomes, with normal proteasomal activity per proteasome. Notably, several downstream targets of the autophagosomal degradation machinery, such as lysosome‐associated membrane protein 2 (LAMP2), BAG3 and SQSTM1, were significantly elevated in MCK‐Cre/cKO muscle lysates to ~160%, ~150% and ~140%, respectively (Figure 3E). Especially, the protein levels of SQSTM1 marked for degradation, denoted by phosphorylation at serine (Ser) 349 or Ser403, were drastically increased in plectin‐deficient muscles to ~200% and ~170% of wild‐type levels (Figure 3E). Quantitative evaluation of lipidated, membrane‐bound LC3‐II (Figure 3F, lower band) versus cytoplasmic LC3‐I form (upper band) [22] revealed that both versions were significantly elevated, to ~140% (LC3‐I) and ~290% (LC3‐II) in MCK‐Cre/cKO muscle lysates, respectively. The ratio of LC3‐II to LC3‐I appeared more than twice as high in plectin‐deficient compared with wild‐type muscles, indicating massively increased levels of membrane‐associated LC3 in MCK‐Cre/cKO samples.

Because of the progressive nature of plectinopathy‐associated muscle degeneration, we assessed whether the observed autophagy‐associated pathological alterations amplify in an age‐dependent manner. In skeletal muscle lysates from 40‐week‐old MCK‐Cre/cKO mice mTOR was decreased to ~70%, while ULK1 and Beclin‐1 were increased to ~150% and ~200% of wild‐type levels, respectively, while protein levels of ATG3, ATG5, and ATG7 remained comparable between both genotypes (Figure S4A). Levels of ubiquitinated proteins (Figure S4B) as well as chymotrypsin‐ and trypsin‐like proteasomal activities were significantly elevated in conjunction with increased protein levels of proteasomal subunits, while caspase‐like proteasomal activities were reduced in aged MCK‐Cre/cKO muscles compared with wild‐type samples (Figure S4C,D). Increased amounts of LAMP2, BAG3, SQSTM1, LC3‐I and LC3‐II in aged MCK‐Cre/cKO muscle lysates (Figure S4E,F) appeared reminiscent of the changes observed in 13‐week‐old animals. Contrary to young mice, the ratio of LC3‐II to LC3‐I was lower in MCK‐Cre/cKO muscles from aged animals compared with their wild‐type counterparts. These analyses suggested that altered autophagic degradation, manifesting as prominent accumulation of autophagy effector proteins while leaving the upstream machineries largely intact, already manifested at a younger age.

### 3.5 Impaired Autophagosome Turnover in Plectin Deficient Myoblasts: Evaluation of Basal Conditions, Inhibition, and Activation of Autophagy

Accumulation of autophagic compartments and downstream target proteins, as observed in plectinopathic muscles, could either result from increased autophagy or impaired later degradation [22]. However, as autophagy is an utterly active process, unravelling autophagic flux in plectin deficiency requires more dynamic examination approaches and the need for corresponding cell culture models. Immortalized *Plec* ^*−/−*^ myoblasts, when differentiated into multinucleated myotubes, reproduced critical pathological alterations of plectinopathic muscle fibres, including Z‐disk aberrations and accumulation of desmin‐positive protein aggregates [10]. On a single cell level, *Plec* ^*−/−*^ myoblasts recapitulated the autophagy‐associated alterations observed in plectinopathy patients and mice, including enrichment of SQSTM1‐positive bodies in the cytoplasm (Figure S5A) and numerous autophagic vacuoles, as observed in electron microscopy (Figure S5B). While mRNA expression levels of *Ulk1, Becn1*, *Lamp2*, *Bag3*, *Sqstm1*, *Map 1lc3a* and *Map 1lc3b* were not altered (Figure S5C), the protein levels of Beclin‐1 were significantly increased (to ~180%) compared with *Plec* ^*+/+*^ cells (Figure S5D). No differences were observed for mTOR or ULK1 protein levels (Figure S5D) or the total amount of polyubiquitinated proteins (Figure S5E); 4‐h or 8‐h treatment with the proteasomal inhibitor MG132 resulted in accumulation of polyubiquitinated proteins in *Plec* ^*+/+*^ and *Plec* ^*−/−*^ myoblasts, even though the increase in ubiquitinated proteins appeared less pronounced in *Plec* ^*−/−*^ cells (Figure S6A). However, while the chymotrypsin‐like proteasomal activity was slightly reduced (Figure S6B,C), the downstream targets of the autophagosomal degradation machinery, e.g., BAG3 and SQSTM1, and especially the Ser349 and Ser403 phosphorylated SQSTM1 versions, were substantially enriched in *Plec* ^*−/−*^ myoblasts (Figure S6D). While the overall LC3 protein levels were reduced, the LC3‐II to LC3‐I ratio displayed a significant increase (to ~140%), indicating that a higher proportion of LC3 was membrane‐associated in *Plec* ^*−/−*^ cells (Figure S6E). Altogether, our experiments confirmed that plectin deficiency evokes striking alterations in the autophagic pathway in humans, mice and myoblasts and demonstrated the applicability of the cell system for further analyses.

**FIGURE 4:**
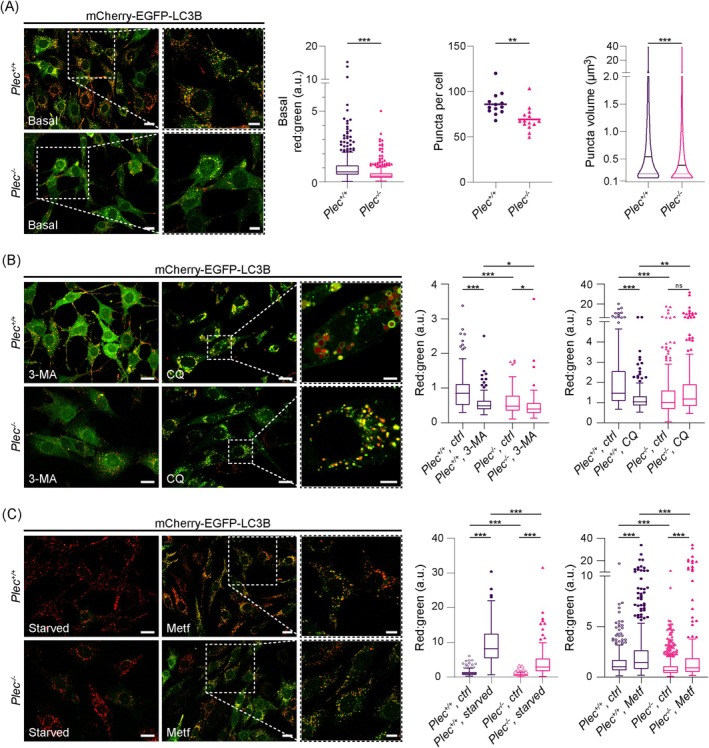
Impaired autophagic flux in mCherry‐EGFP‐LC3B‐expressing plectin‐deficient myoblast cell lines. (A) Immortalized (*p53* ^*−/−*^) plectin‐expressing (*Plec* ^*+/+*^) and plectin‐deficient (*Plec* ^*−/−*^) myoblasts stably expressing mCherry‐EGFP‐LC3B at basal conditions. Panels on the right are single plane magnifications of the boxed areas indicated in the confocal images in the panels on the left. Note increased cytoplasmic LC3B signals *Plec* ^*−/−*^ myoblasts. Scale bars: 20 μm, magnifications 10 μm. Red:green signal ratios of mCherry‐EGFP‐LC3B‐expressing *Plec* ^*+/+*^ and *Plec* ^*−/−*^ myoblasts at basal conditions: box plots show the median and Tukey whiskers (*Plec* ^*+/+*^, *n* = 468 cells; *Plec* ^*−/−*^, *n* = 455 cells); ***p < 0.001 (two‐tailed Mann–Whitney test). Number of mCherry‐positive puncta per cell was determined by using the 3D Objects Counter; each dot represents a single field‐of‐view, the line represents the mean (*Plec* ^*+/+*^, *n* = 13 field‐of‐views; *Plec* ^*−/−*^, *n* = 14 field‐of‐views); **p < 0.01 (two‐tailed, unpaired *t* test with Welch's correction). Volume of mCherry‐positive puncta was determined by using the 3D Objects Counter; violin plots show median, the 25^th^ and 75^th^ percentile (*Plec* ^*+/+*^, *n* = 38 926 puncta [468 cells]; *Plec* ^*−/−*^, *n* = 29 302 puncta [455 cells]); ***p < 0.001 (two‐tailed Mann–Whitney test). (B) mCherry‐EGFP‐LC3B‐expressing *Plec* ^*+/+*^ and *Plec* ^*−/−*^ myoblasts were treated with 9‐mM 3‐methyladenine (3‐MA) or 50‐μM chloroquine (CQ) for 3 h. Control cells (ctrl) were either kept in DMEM‐based growth medium containing 10% FCS (3‐MA treatment) or in F‐10‐based growth medium containing 20% FCS (CQ treatment), as described in the Supporting Information. Panels on the right are single plane magnifications of the boxed areas indicated in the confocal images in the centre panels. Note the increased cytoplasmic LC3B signals upon 3‐MA treatment, and the massive swelling of vesicles upon CQ treatment in *Plec* ^*+/+*^ myoblasts, while *Plec* ^*−/−*^ myoblasts remained largely unaltered. Also note the occurrence of partially acidified autolysosomes in CQ‐treated *Plec* ^*+/+*^ myoblasts, as denoted by the formation of green ring‐like structures. Scale bars: 20 μm, magnifications 5 μm. Red:green signal ratios of ctrl‐, 3‐MA‐ and CQ‐treated mCherry‐EGFP‐LC3B‐expressing *Plec* ^*+/+*^ and *Plec* ^*−/−*^ myoblasts: box plots show the median and Tukey whiskers (*Plec* ^*+/+*^, *n* = 103/113 [control/3‐MA] and 202/260 [control/CQ] cells; *Plec* ^*−/−*^, *n* = 89/81 [control/3‐MA] and 142/206 [control/CQ] cells); *p < 0.05, **p < 0.01, ***p < 0.001 (two‐way ANOVA of the ranked dataset with Tukey's post hoc correction for multiple comparisons); ns, not significant. (C) mCherry‐EGFP‐LC3B‐expressing *Plec* ^*+/+*^ and *Plec* ^*−/−*^ myoblasts were starved for 24 h or treated with 100‐mM metformin (Metf) for 48 h. Ctrl cells were either kept in DMEM‐based growth medium containing 10% FCS (starvation) or in F‐10‐based growth medium containing 20% FCS (Metf treatment). Panels on the right are single plane magnifications of the boxed areas indicated in the confocal images in the centre panels. Note the increased red signals in both cell lines compared with control conditions. Scale bars: 20 μm, magnifications 10 μm. Red:green signal ratios of ctrl‐, starved or Metf‐treated mCherry‐EGFP‐LC3B‐expressing *Plec* ^*+/+*^ and *Plec* ^*−/−*^ myoblasts: box plots show the median and Tukey whiskers (*Plec* ^*+/+*^, *n* = 185/177 [control/starved] and 500/460 [control/Metf] cells; *Plec* ^*−/−*^, *n* = 176/177 [control/starved] and 630/285 [control/Metf] cells). ***p < 0.001 (two‐way ANOVA of the ranked dataset with Tukey's post hoc correction for multiple comparisons).

Next, we generated *Plec* ^*+/+*^ and *Plec* ^*−/−*^ myoblast cell lines stably expressing a mCherry‐EGFP‐LC3B reporter [15] by retroviral transduction. In these cells, autophagic flux can be determined due to the quenching of EGFP in the acidic lysosome, whereas the more stable mCherry remains preserved, thereby enabling us to follow the transitions of autophagic compartments from autophagosomes (yellow) to autolysosomes (red); accordingly, the red:green signal ratio is considered a measure of autophagic flux [23]. At basal conditions, mCherry‐EGFP‐LC3B‐expressing *Plec* ^*+/+*^ myoblasts displayed distinct puncta formation in the reddish spectrum, while plectin‐deficient cells harboured primarily green puncta as well as diffuse cytoplasmic EGFP‐LC3B signals. *Plec* ^*−/−*^ myoblasts displayed a significantly reduced mean red:green ratio of ~0.4 (*Plec* ^*+/+*^: ~0.7), had only ~69 puncta per cell (*Plec* ^*+/+*^: ~86), and the puncta were smaller, i.e., a reduced median puncta volume of ~0.39 μm^3^ in *Plec* ^*−/−*^ myoblasts (diameter [*d*] = 0.90 μm; *Plec* ^*+/+*^: ~0.54 μm^3^, corresponding to *d* * *a* = 1.00 μm, assumed as a perfect sphere; Figure 4A). Together, these data pointed towards diminished formation and volume of autophagic compartments in mCherry‐EGFP‐LC3B‐expressing *Plec* ^*−/−*^ myoblasts at basal conditions.

To explore the effects of impaired autophagic flux, cells were next treated with commonly used inhibitors of autophagy (Figure 4B). Application of 3‐methyladenine (3‐MA), an inhibitor of autophagic sequestration, for 3 h, led to enhanced cytoplasmic signals in mCherry‐EGFP‐LC3B‐expressing *Plec* ^*+/+*^ myoblasts and a significant drop in the signal ratios to ~40% of untreated controls. In contrast, the subcellular distribution of mCherry‐EGFP‐LC3B in 3‐MA‐treated *Plec* ^*−/−*^ myoblasts remained largely unaltered and the signal ratios were slightly reduced to ~80% of untreated controls. Inhibition of late‐stage autophagy by application of CQ for 3 h caused swollen and ballooned autophagic vacuoles in *Plec* ^*+/+*^ cells; defective degradation was emphasized by the occurrence of partially acidified vesicles (Figure 4B, magnifications), accompanied by significantly reduced red:green ratios (*Plec* ^*+/+*^: ~1.5 at control conditions vs. ~1.0 after CQ treatment). Notably, CQ‐treated *Plec* ^*−/−*^ cells appeared similar to untreated *Plec* ^*−/−*^ controls and the red:green ratios remained unaffected (*Plec* ^*−/−*^: ~1.2 at control conditions vs. ~1.2 after CQ treatment). Treatment with bafilomycin A1 (Baf A1), inhibiting the autophagosome/lysosome fusion, had similar effects as the CQ treatment (Figure S7A). Long‐term 24‐h application of CQ or Baf A1 led to almost exclusive bright yellow signals as well as comparable red:green signal ratios in mCherry‐EGFP‐LC3B‐expressing *Plec* ^*+/+*^ and *Plec* ^*−/−*^ cells (Figure S7B), indicating an indistinguishable accumulation of autophagic vesicles after elongated inhibition. 24 h treatment with 3‐MA, on the other hand, caused a return to baseline levels (Figure S7C).

To actively trigger autophagy, cells were either starved or treated with metformin (Metf) (Figure 4C). While 3‐h starvation slightly activated autophagy (Figure S7D), 24‐h starvation led to bright red signals in both genotypes and a ~8‐ and ~6‐fold increase in red:green ratios for *Plec* ^*+/+*^ and *Plec* ^*−/−*^ cells, respectively; 48‐h Metf treatment, though less invasive, also triggered autophagic turnover, as illustrated by distinct puncta formation (Figure 4C, magnifications). Notably, the median red:green ratio for Metf‐treated *Plec* ^*−/−*^ myoblasts (~0.9) was comparable to that for untreated *Plec* ^*+/+*^ cells (~1.0; *p* = 0.34). Collectively, our experiments indicated significantly compromised autophagic flux in mCherry‐EGFP‐LC3B‐expressing *Plec* ^*−/−*^ myoblasts.

**FIGURE 5:**
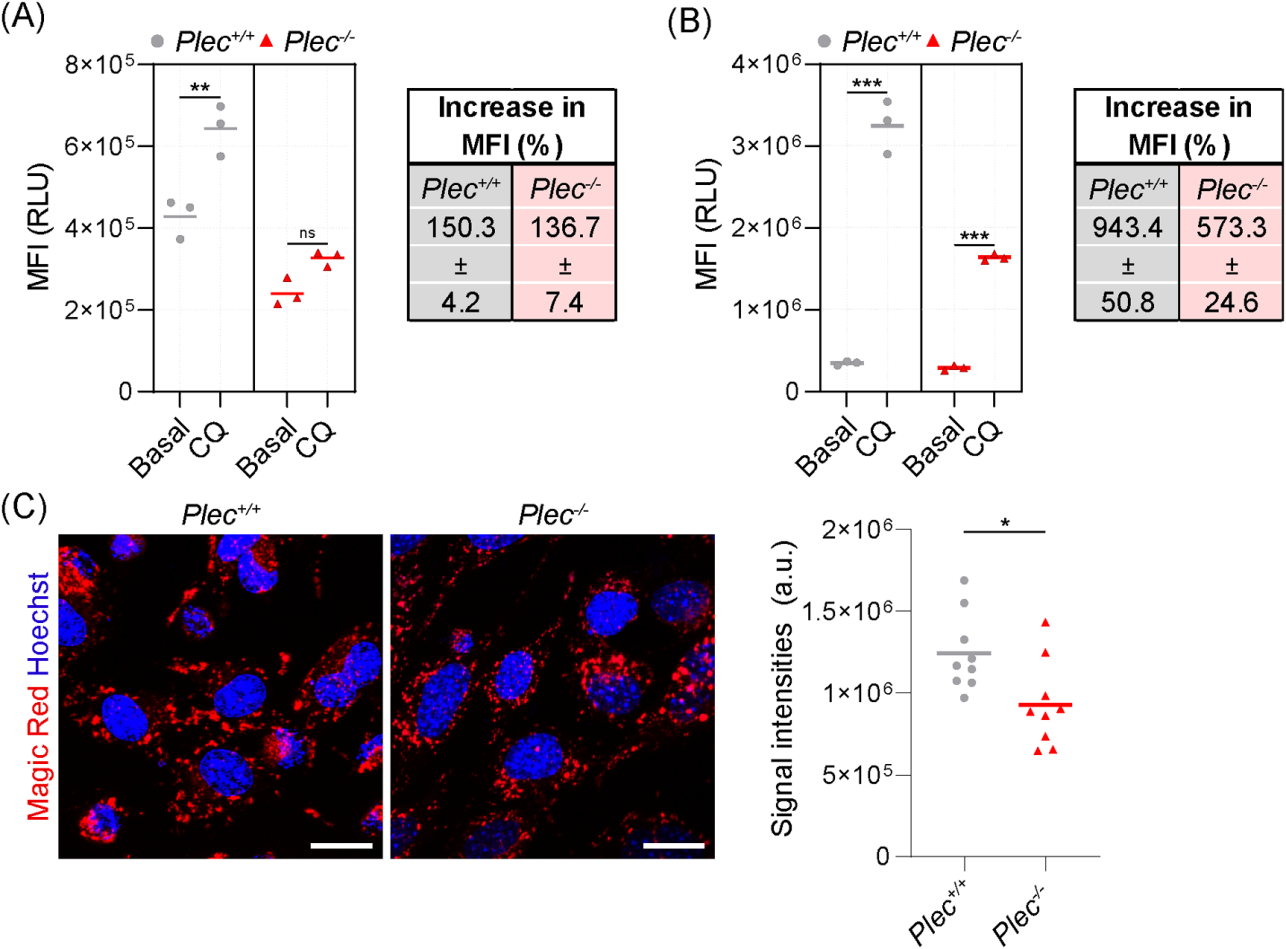
Impaired autophagic flux, reduced lysosomal capacities and reduced intracellular cathepsin B protease activity in *Plec* ^*−/−*^ myoblasts. (A) Flow cytometry of CYTO‐ID‐stained *Plec* ^*+/+*^ and *Plec* ^*−/−*^ myoblasts at control conditions or treated with 50‐μM CQ for 3 h and calculation of the increase in median fluorescence intensity (MFI) upon CQ treatment (CQ:control). Line shows the mean, *n* = 3 experiments (7.5 × 10^4^–9.5 × 10^4^ cells/experiment); RLU, relative light units. (B) Flow cytometry of LYSO‐ID‐stained *Plec* ^*+/+*^ and *Plec* ^*−/−*^ myoblasts at control conditions or treated with 50‐μM CQ for 3‐h calculation of the increase MFI upon CQ treatment (CQ:control). Line shows the mean; *n* = 3 experiments (4.1 × 10^4^–9.4 × 10^4^ cells/experiment). (C) *Plec* ^*+/+*^ and *Plec* ^*−/−*^ myoblasts were stained with the Magic Red Cathepsin B Assay Kit. Nuclei were visualized with Hoechst. Scale bars: 20 μm. Magic Red signal intensities were calculated by normalizing the RawIntDens of a field‐of‐view to the number of cells. Each dot represents a single field‐of‐view, the line represents the mean (*Plec* ^*+/+*^, *n* = 9 [723 cells]; *Plec* ^*−/−*^, *n* = 9 [748 cells] field‐of‐views). For A–C: *p < 0.05, **p < 0.01, ***p < 0.001 (two‐tailed, unpaired *t* test with Welch's correction); ns, not significant.

In addition, we used the cationic amphiphilic tracer dye CYTO‐ID, comprehensively labelling autophagic compartments [24], and quantified autophagosomal staining in *Plec* ^*+/+*^ and *Plec* ^*−/−*^ myoblasts cell lines using flow cytometry (Figure 5A). As expected, the average median fluorescence intensity (MFI) of *Plec* ^*−/−*^ myoblasts was significantly reduced to ~3.3 × 10^5^ (*Plec* ^*+/+*^: ~4.8 × 10^5^). Upon CQ treatment, the MFI of *Plec* ^*+/+*^ myoblasts increased to ~150%, while the MFI of CQ‐treated *Plec* ^*−/−*^ cells only increased to ~137% compared with the respective controls, indicating a lower response. To address the question of whether the downstream clearance was also affected in plectin‐deficient cells, acidic compartments such as endosomes, lysosomes and autolysosomes were investigated by flow cytometry of LYSO‐ID‐stained myoblasts (Figure 5B). Here, basal conditions revealed a trend towards a ~20% reduced labelling in *Plec* ^*−/−*^ myoblasts. In CQ‐treated *Plec* ^*+/+*^ myoblasts, the MFI increased to ~940%, while *Plec* ^*−/−*^ cells reached only a MFI increase of ~570% upon CQ treatment, pointing towards explicit differences between the two genotypes. Finally, lysosomal degradation capacities were assessed by measuring cathepsin B activity in *Plec* ^*+/+*^ and *Plec* ^*−/−*^ myoblasts (Figure 5C). Notably, the Magic Red signal intensities were significantly decreased in *Plec* ^*−/−*^ myoblasts, proving reduced levels of cathepsin B‐mediated proteolysis, i.e., diminished functional lysosomes in plectin‐deficient cells.

### 3.6 Impaired Autophagic Flux and Reduced Lysosomal Capacities in Primary Plectin‐Deficient Myoblasts and Fibroblasts

**FIGURE 6:**
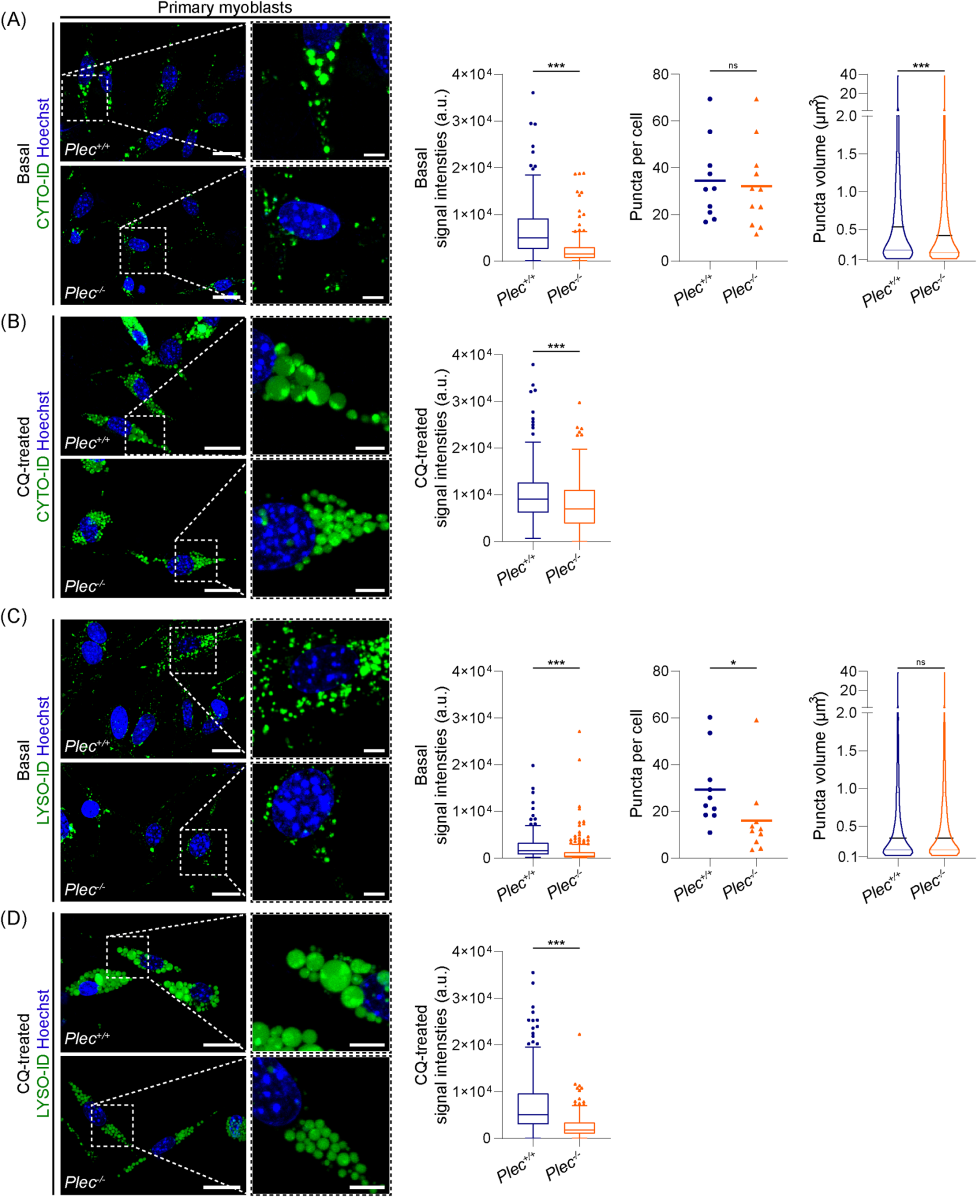
Impaired autophagic flux and reduced capacities of acidic compartments in primary plectin‐deficient myoblasts. (A) Primary murine plectin‐expressing myoblasts (*Plec* ^*+/+*^) or plectin‐deficient (*Plec* ^*−/−*^) myoblasts were stained with CYTO‐ID at control conditions. Nuclei were visualized with Hoechst. Panels on the right are magnifications of the boxed areas indicated in the panels on the left. Scale bars: 20 μm, magnifications 5 μm. CYTO‐ID signal intensities in *Plec* ^*+/+*^ and *Plec* ^*−/−*^ myoblasts at control conditions were calculated by normalizing the RawIntDens to cell areas. Box plots show the median and Tukey whiskers (*Plec* ^*+/+*^, *n* = 177 cells; *Plec* ^*−/−*^, *n* = 145 cells). Number of CYTO‐ID‐positive puncta per cell was determined by using the 3D Objects Counter. Each dot represents a single field‐of‐view, the line represents the mean (*Plec* ^*+/+*^, *n* = 10 field‐of‐views; *Plec* ^*−/−*^, *n* = 14 field‐of‐views). Volume of CYTO‐ID‐positive puncta was determined by using the 3D Objects Counter. Violin plots show median, the 25^th^ and 75^th^ percentile (*Plec* ^*+/+*^, *n* = 7005 puncta [177 cells]; *Plec* ^*−/−*^, *n* = 4474 puncta [145 cells]). (B) Primary *Plec* ^*+/+*^ and *Plec* ^*−/−*^ myoblasts were treated with 50‐μM CQ for 3 h and stained with CYTO‐ID. Nuclei were visualized with Hoechst. Panels on the right are magnifications of the boxed areas indicated in the panels on the left. Note the massively swollen vesicles in CQ‐treated *Plec* ^*+/+*^ myoblasts, while CQ treatment of *Plec* ^*−/−*^ myoblasts caused marginal vesicle swelling. Scale bars: 20 μm, 5‐μm magnifications. CYTO‐ID signal intensities in CQ‐treated *Plec* ^*+/+*^ and *Plec* ^*−/−*^ myoblasts were calculated by normalizing the RawIntDens to cell areas. Box plots show the median and Tukey whiskers (*Plec* ^*+/+*^, *n* = 187 cells; *Plec* ^*−/−*^, *n* = 172 cells). (C) Primary *Plec* ^*+/+*^ and *Plec* ^*−/−*^ myoblasts were stained with LYSO‐ID at control conditions. Nuclei were visualized with Hoechst. Panels on the right are magnifications of the boxed areas indicated in the panels on the left. Scale bars: 20 μm, magnifications 5 μm. LYSO‐ID signal intensities in *Plec* ^*+/+*^ and *Plec* ^*−/−*^ myoblasts at control conditions were calculated by normalizing the RawIntDens to cell areas. Box plots show the median and Tukey whiskers (*Plec* ^*+/+*^, *n* = 166 cells; *Plec* ^*−/−*^, *n* = 155 cells). Number of LYSO‐ID‐positive puncta per cell was determined by using the 3D Objects Counter. Each dot represents a single field‐of‐view, the line represents the mean (*Plec* ^*+/+*^, *n* = 10 field‐of‐views; *Plec* ^*−/−*^, *n* = 10 field‐of‐views). Volume of LYSO‐ID‐positive puncta was determined by using the 3D Objects Counter. Violin plots show median, the 25^th^ and 75^th^ percentile (*Plec* ^*+/+*^, *n* = 4574 puncta [166 cells]; *Plec* ^*−/−*^, *n* = 2452 puncta [155 cells]). (D) Primary *Plec* ^*+/+*^ and *Plec* ^*−/−*^ myoblasts were treated with 50‐μM CQ for 3 h and stained with LYSO‐ID. Nuclei were visualized with Hoechst. Panels on the right are magnifications of the boxed areas indicated in the panels on the left. Note the massively swollen vesicles in CQ‐treated *Plec* ^*+/+*^ myoblasts, while CQ treatment of *Plec* ^*−/−*^ myoblasts caused marginal vesicle swelling. Scale bars: 20 μm, magnifications 5 μm. LYSO‐ID signal intensities in CQ‐treated *Plec* ^*+/+*^ and *Plec* ^*−/−*^ were calculated by normalizing the RawIntDens to cell areas. Box plots show the median and Tukey whiskers (*Plec* ^*+/+*^, *n* = 246; *Plec* ^*−/−*^, *n* = 186 cells). For A–D: *p < 0.05, ***p < 0.001 (two‐tailed Mann–Whitney test); ns, not significant.

As the immortalized cell lines were derived from *p53* ^*−/−*^ mice, our plectinopathy cell models represent double knockout systems; i.e., they lack p53 in addition to plectin [10]. Thus, phenotypes observed on the molecular or cellular level, attributed to the lack of plectin, could also (at least in part) be due to p53 deficiency. To establish the specificity of the phenotypic traits, key observations were confirmed in primary myoblast cultures derived from normal (not *p53* ^*−/−*^) neonatal *Plec* ^*+/+*^ and *Plec* ^*−/−*^ mice [25]. When primary *Plec* ^*−/−*^ myoblasts were analysed using the CYTO‐ID dye by life‐cell imaging (Figure 6A), their autophagic vesicles appeared smaller than in primary *Plec* ^*+/+*^ cells and the median CYTO‐ID signal intensities were significantly decreased to ~1.6 × 10^3^ (*Plec* ^*+/+*^: ~5.0 × 10^3^). Notably, while both genotypes harboured a similar number of puncta per cell (primary *Plec* ^*+/+*^: ~34, *Plec* ^*−/−*^: ~32), the median puncta volume was significantly reduced to ~0.42 μm^3^ (*d* = 0.93 μm) in primary *Plec* ^*−/−*^ myoblasts (*Plec* ^*+/+*^: ~0.54 μm^3^, *d* = 1.00 μm), suggesting that smaller puncta were the cause of the reduced CYTO‐ID signal intensities. CQ treatment led to a drastic swelling of autophagic vacuoles in primary *Plec* ^*+/+*^ myoblasts, while the swelling in plectin‐deficient cells was moderate (Figure 6B). This was also reflected by the ~1.3‐fold higher CYTO‐ID signal intensities in primary *Plec* ^*+/+*^ versus *Plec* ^*−/−*^ myoblasts. LYSO‐ID staining of *Plec* ^*−/−*^ myoblasts revealed a significant drop in the basal fluorescence intensities (~60% of *Plec* ^*+/+*^ levels), and a reduction to only ~19 puncta per *Plec* ^*−/−*^ myoblast (*Plec* ^*+/+*^: ~30 puncta). Because the median puncta volume remained similar in both genotypes, reduced signal intensities correlated to a reduced number of acidic compartments. Consistently, application of CQ resulted in less vesicle swelling in primary *Plec* ^*−/−*^ myoblasts, coinciding with a reduced median signal intensity (*Plec* ^*−/−*^: ~1.8 × 10^3^, *Plec* ^*+/+*^: ~5.0 × 10^3^; Figure 6D). Together, our dynamic monitoring of immortalized and primary plectin‐deficient myoblasts unequivocally established a defect in their autophagic flux and lysosomal degradation.

**FIGURE 7:**
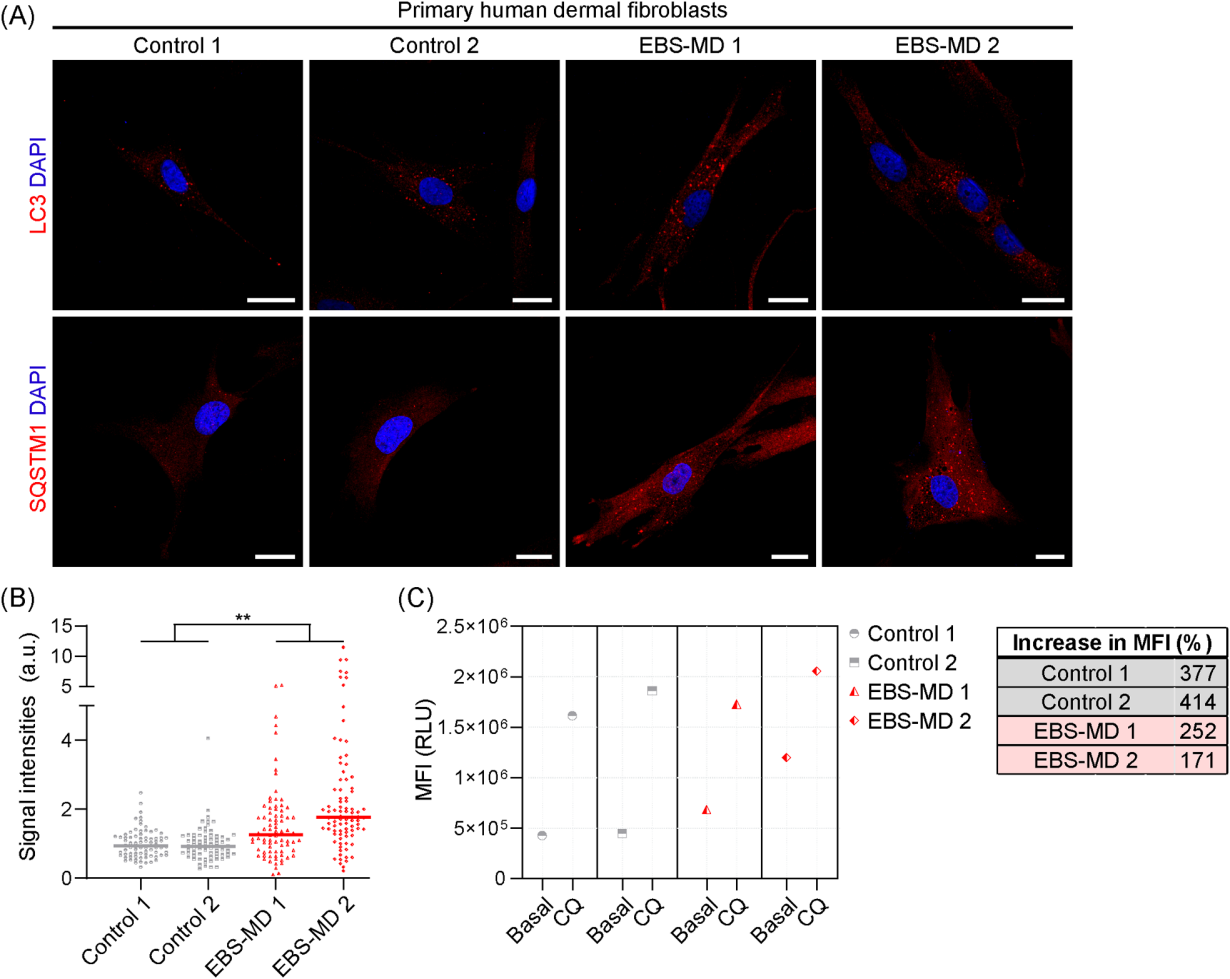
Reduced autophagic capacities in EBS‐MD patient fibroblasts. (A) Immunostaining of primary human dermal fibroblasts obtained from healthy controls or EBS‐MD patients using antibodies to LC3 or SQSMT1. Nuclei were visualized with DAPI. Scale bars: 20 μm. (B) SQSTM1 signal intensities in control and EBS‐MD fibroblasts were calculated by normalizing the RawIntDens to cell areas. Each dot represents a single cell, the line represents the median (control 2, *n* = 78 cells; control 3, *n* = 73 cells; EBS‐MD 4, *n* = 82 cells; EBS‐MD 5, *n* = 88 cells); **p < 0.01 (Kruskal–Wallis test with Dunn's correction for multiple comparisons). (C) Flow cytometry of CYTO‐ID‐stained control and EBS‐MD fibroblasts at control conditions or treated with 50‐μM CQ for 3 h and calculation of the increase in MFI upon treatment with CQ (CQ:control). Each dot represents the MFI (control 2, *n* = 8.3 × 10^4^/8.5 × 10^4^ [control/CQ treated] cells; control 3, *n* = 7.8 × 10^4^/6.8 × 10^4^ [control/CQ treated] cells; EBS‐MD 1, *n* = 8.2 × 10^4^/7.2 × 10^4^ [control/CQ treated] cells; EBS‐MD 2, *n* = 7.5 × 10^4^/4.4 × 10^4^ [control/CQ treated] cells).

To recapitulate the apparent block in autophagy in patient‐derived cells, primary human dermal fibroblasts from two EBS‐MD patients [17, 18, 19] as well as from two healthy controls were immunolabelled for LC3 or SQSTM1 (Figure 7A). Both autophagy marker proteins were clearly enhanced in EBS‐MD fibroblasts, with relative SQSTM1signal intensities significantly elevated in EBS‐MD fibroblasts 1 and 2 to ~140% and ~190%, respectively, of control levels (Figure 7B). Flow cytometric analyses of CYTO‐ID‐labelled human fibroblasts at basal conditions revealed markedly increased MFIs of EBS‐MD 1 and 2 to ~6.8 × 10^5^ and ~1.2 × 10^6^, respectively (control 1: ~4.3 × 10^5^, control 2: ~4.5 × 10^5^; Figure 7C). Incubation with CQ for 3 h increased MFIs to comparable levels in EBS‐MD and control fibroblasts, which is a significantly lower proportional change in MFIs in EBS‐MD fibroblasts compared with control cells, confirming that plectin deficiency coincides with impaired autophagic turnover, not only in myoblasts, but also in other cell types.

### 3.7 Compromised Autophagic Flux in Plectin‐Deficient Skeletal Muscle

Having identified impaired autophagic flux in plectin‐deficient myoblasts, we aimed at consolidating this observation in mice. Accordingly, autophagy was inhibited in wild‐type and MCK‐Cre/cKO mice by intraperitoneal application of CQ for 4 h [14], and frozen muscles were subsequently evaluated. In CQ‐treated wild‐type muscles we identified multiple fibres with massively enhanced SQSTM1 signals compared with saline‐treated controls, indicating accumulation of downstream substrates upon inhibition of autophagy (Figure S8A). Contrary, even though increased SQSTM1 staining pattern was already evident in saline‐treated MCK‐Cre/cKO muscles, CQ treatment did not aggravate the pathology. In keeping with this finding, evaluation of BAG3, SQSTM1, Ser403 phosphorylated SQSTM1, LC3‐I and LC3‐II revealed an overall trend towards increased autophagy marker proteins in CQ‐treated versus saline‐treated wild‐type muscles, while CQ‐treated MCK‐Cre/cKO muscles remained comparable to saline‐treated controls (Figure S8B;C). Ultrastructural changes in CQ‐treated wild‐type samples included numerous swollen vacuoles and membrane whirls, while CQ‐treated MCK‐Cre/cKO samples showed similar results as saline‐treated controls (Figure S8D). In addition, when accumulation of lysosomes was assessed by acid phosphatase enzymatic reactions on muscle sections and AI‐based evaluation, relative mean intensities significantly increased in CQ‐treated versus saline‐treated wild‐type muscles (saline‐treated: ~0.75, CQ‐treated: ~0.79; Figure S8E), while CQ treatment did not shift signals in MCK‐Cre/cKO myofibres (saline‐treated: ~0.82, CQ‐treated: ~0.82). Taken together, autophagy inhibition by CQ treatment had only tenuous effects on the autophagy‐associated pathology in MCK‐Cre/cKO, reassuring in vivo the concept that autophagic flux is impaired in plectin‐deficient muscle.

### 3.8 Metformin Treatment In Vivo: Therapeutic Perspectives for Plectinopathies?

**FIGURE 8:**
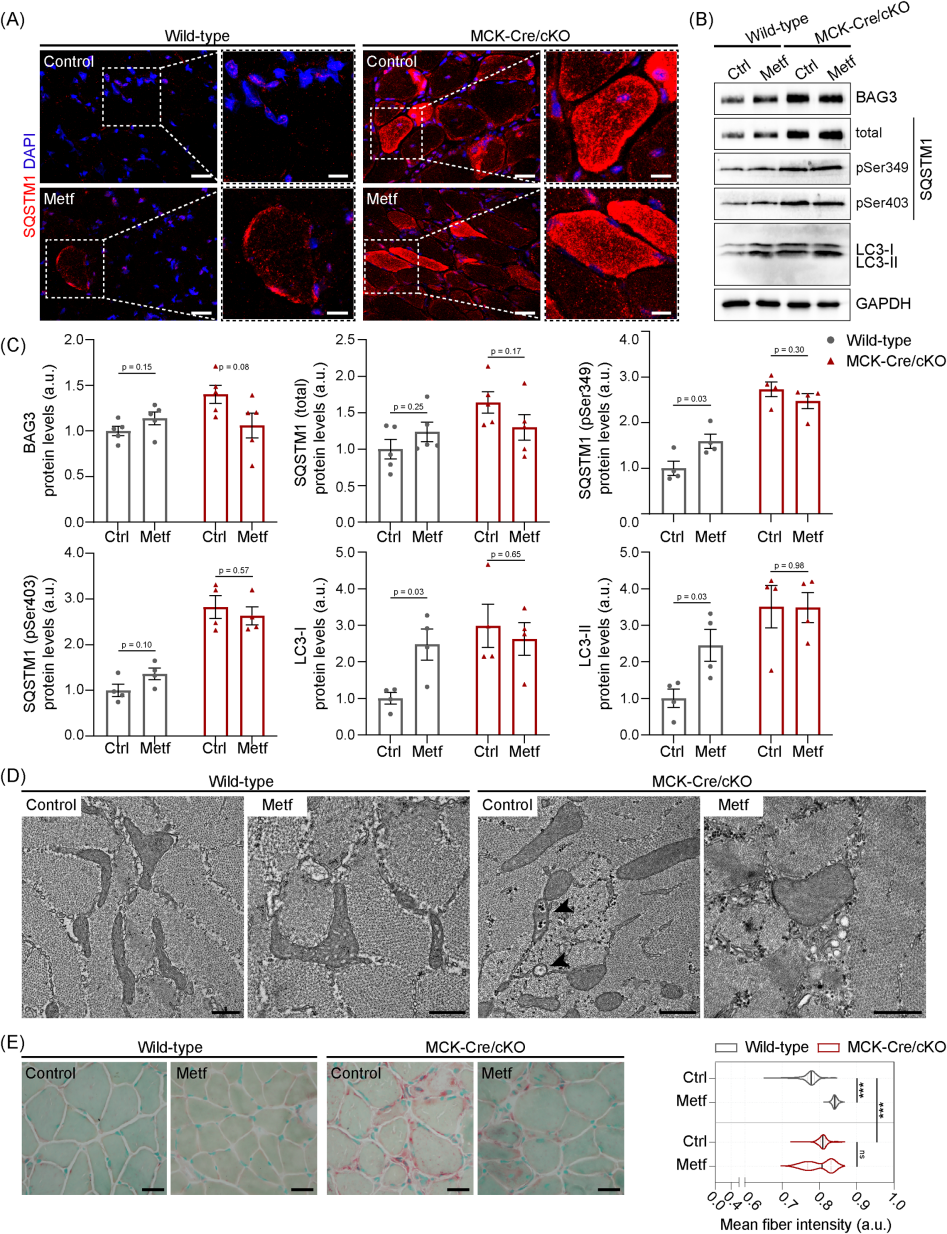
Metformin treatment of wild‐type and MCK‐Cre/cKO mice. (A) Immunostaining using antibodies to SQSTM1 of frozen muscle sections prepared from wild‐type and MCK‐Cre/cKO mice treated for 30 days with sweetened water with or without 500 mg/kg/day Metf. Nuclei were visualized with DAPI. Panels on the right are magnifications of the boxed areas indicated in the panels on the left. Note enhanced SQSTM1 signals in wild‐type muscles upon Metf‐treatment, whereas the SQSTM1 signals in MCK‐Cre/cKO muscles remain similar to control (Ctrl)‐treated samples. Scale bars: 20 μm, magnifications 10 μm. (B) Immunoblotting of Ctrl‐ or Metf‐treated wild‐type and MCK‐Cre/cKO muscle lysates using antibodies to BAG3, total and phosphorylated forms of SQSTM1, LC3, and GAPDH. (C) Signal intensities of protein bands as shown in (B) were densitometrically measured and normalized to the total protein content as analysed by Coomassie staining (not shown). Mean ± SEM; *n* = 3–5; all *p* values are indicated (unpaired, two‐tailed *t* test with Welch's correction). (D) Representative electron micrographs of soleus muscle cross sections obtained from Ctrl‐ or Metf‐treated wild‐type and MCK‐Cre/cKO mice. Note the occurrence of mitochondrial abnormalities and degradative vacuoles (arrowheads) in Ctrl‐treated MCK‐Cre/cKO muscles; no aggravation of autophagolytic changes was observed upon Metf‐treatment. Scale bars: 500 nm. (E) Soleus muscle sections from Ctrl‐ or Metf‐treated wild‐type and MCK‐Cre/cKO mice were stained with acid phosphatase (AP) and subjected to AI‐based evaluation of AP signal intensities within individual myofibres using MIRA Vision. Scale bars: 25 μm. Note the increased AP staining (red) within Metf‐treated wild‐type, as well as Ctrl‐ and Metf‐treated MCK‐Cre/cKO myofibres. Also note the switch in mean fibre intensity distribution in MCK‐Cre/cKO muscles from a normal distribution (Ctrl‐treated) to bimodal (Metf‐treated). Wild‐type, *n* = 1491/1295 fibres [Ctrl/Metf]; MCK‐Cre/cKO, *n* = 1456/1287 fibres [Ctrl/Metf]; two animals each; ***p < 0.001 (two‐way ANOVA of the ranked dataset with Tukey's post hoc correction for multiple comparisons); ns, not significant.

Even though induction of autophagic flux by Metf was less efficient in *Plec* ^*−/−*^ myoblasts compared with controls (see Figure 4C), it was nevertheless partially rescued. Accordingly, we aimed at investigating whether application of Metf in vivo might ameliorate the observed phenotype in mice and treated wild‐type and MCK‐Cre/cKO mice with Metf for 30 days. Frozen soleus muscles of Metf‐treated wild‐type muscles displayed fibres with enhanced SQSTM1 signals compared with controls, indicating activation of autophagy, while Metf treatment did not aggravate the pathology of MCK‐Cre/cKO muscles (Figure 8A). Accordingly, significantly enhanced levels of LC3‐I and LC3‐II as well as trends towards increased protein levels of BAG3, SQSTM1, Ser349 and Ser403 phosphorylated SQSTM1 marker proteins were noted in Metf‐treated versus control‐treated wild‐type lower leg muscle lysates (Figure 8B,C), while the levels of autophagy marker proteins remained comparable in Metf‐treated and control‐treated MCK‐Cre/cKO muscle lysates or even displayed a trend towards decreased protein levels. Electron microscopy of soleus muscle cross sections revealed the occurrence of mitochondrial abnormalities and degradative vacuoles in control‐treated MCK‐Cre/cKO muscles; however, no aggravation of autophagolytic changes was observed upon Metf treatment (Figure 8D). In keeping with the previous findings, acid phosphatase enzymatic reactions and AI‐based evaluation unravelled significantly increased relative mean intensities in Metf‐treated versus control‐treated wild‐type muscles (control‐treated: ~0.77, Metf‐treated: ~0.84; Figure 8E). Notably, while the relative mean intensities were comparable in Metf‐treated versus control‐treated MCK‐Cre/cKO muscle fibres (control‐treated: ~0.81, Metf‐treated: ~0.79), the distribution of Metf‐treated fibre intensities shifted from normal distribution to bimodal, indicating a marked reaction to the treatment and interfibre variability. Thus, activating autophagy by Metf might have a positive effect, leading to an amelioration of the autophagic dysfunction in plectin deficiency.

## 4 Discussion

The present work demonstrated that the characteristic desmin protein aggregation pathology in plectin‐deficient skeletal muscles and cells is associated with major alterations in protein quality control pathways. As alterations in virtually every step of the autophagic cascade can contribute to disease processes in myopathies [26], we elaborately explored autophagy‐relevant pathways. Our experiments revealed that plectin‐deficient muscles significantly accumulate downstream autophagic effector proteins, while leaving the composition of the autophagic machinery largely intact. Because the observed alterations were already present in muscles from young MCK‐Cre/cKO mice, these changes are likely contributing to the early pathology of this progressive and devastating muscle disease. At least in part, our results are in line with studies on muscle‐specific ATG5 or ATG7 knockout mice showing that muscles that are incapable of performing autophagy develop functional and morphological alterations, including muscle loss, protein aggregates, misalignment of Z‐lines, and accumulation of abnormal mitochondria [27, 28]. In addition, loss of autophagy in both studies triggered a compensatory increase in the ubiquitin‐proteasome degradation pathway [27, 28], similar to the increased levels of ubiquitinated proteins and proteasomal activities we observed in MCK‐Cre/cKO muscles. Age‐dependent reduction in proteasomal activities and aggravation of autophagic defects might contribute to disease progression as well.

Because autophagy is a highly dynamic process, analyses on the tissue level only allow a steady assessment of the pathway intermediates without measuring their rate of turnover and degradation, i.e., increased numbers of autophagosomes do not inevitably correspond to increased autophagic activity, but might rather stem from suppressed steps in the autophagic pathway downstream of autophagosome formation [22]. As the exposure to inhibiting or activating conditions is an essential prerequisite for conclusively monitoring autophagic flux [23, 29], we measured baseline, impaired (3‐MA, CQ, or Baf A1 treatments) or enhanced autophagy (starvation or Metf treatment) in plectin‐deficient myoblasts. Notably, mCherry‐EGFP‐LC3B‐expressing *Plec* ^*−/−*^ myoblasts exhibited explicit signs of reduced autophagic flux, i.e., they did rarely respond to the different inhibitors as red:green signal ratios barely dropped. However, long‐term impairment of autophagy revealed bright yellow signals, indicating that *Plec* ^*−/−*^ myoblasts were in principle capable of performing mCherry‐EGFP‐LC3B turnover, albeit at a significantly lower rate than *Plec* ^*+/+*^ cells, and activation of autophagic flux by starvation or Metf treatment was more efficient in *Plec* ^*+/+*^ than in *Plec* ^*−/−*^ myoblasts. Dynamic evaluation of CYTO‐ID‐, LYSO‐ID‐ and/or Magic Red‐stained primary and immortalized plectin‐deficient myoblasts and EBS‐MD patient‐derived fibroblasts further substantiated that loss of plectin unequivocally provokes a dysfunction in the autophagosomal‐lysosomal turnover on the cellular level. A pharmacological blockade of autophagic flux in vivo indicated that only wild‐type muscles accumulated downstream substrates, while in MCK‐Cre/cKO muscles, albeit already displaying hallmarks of increased autophagy, application of CQ did not aggravate the observed pathology. Ultimately, wild‐type and MCK‐Cre/cKO muscles reacted differently to an in vivo treatment with the autophagy activator Metf, opening a perspective for future therapeutic studies. However, whether Metf treatment might improve other EBS‐MD key pathologies, including the accumulation of desmin‐positive protein aggregates in muscle fibres, or even ameliorate muscle function, remains to be investigated. In conclusion, our study convincingly demonstrated that loss of plectin leads to markedly reduced autophagic clearance capacities in skeletal muscle.

Plectin's most prominent role is obviously the mechanical stabilization, interconnection, and recruitment of various types of IFs [2], as highlighted by several studies demonstrating increased bundling or even complete collapse of keratin, vimentin, or desmin IF networks in murine *Plec* ^*−/−*^ keratinocytes, fibroblasts, or myoblasts, respectively [10, 30, 31]. Plectin‐mediated connection between keratin 8 (KRT8) and actin or mitochondria was implicated for correct autophagy and mitophagy, respectively, under oxidative stress in retinal pigment epithelial cells [32, 33]. Intact vimentin networks were prerequisite for autophagosome and lysosome positioning in HEK cells [34], and probably regulate mTORC1 signalling by facilitating its localization to lysosomes [35]. While a direct role for desmin IFs in autophagic transport has not yet been established, desmin deficiency as well as desminopathy‐causing mutations lead to imbalanced protein homeostasis in murine muscles [36, 37]. Furthermore, the mere expression of various desmin mutants impacts autophagic flux in C2C12 myoblasts [38] and desminopathy patients with autophagic vacuolar myopathies have been reported [39]. In the case of plectinopathies, our analyses suggest that the characteristic accumulation and aggregation of desmin in *PLEC* mutant skeletal muscle cells and tissues results from an impaired autophagic turnover. In conclusion, our study opens a new perspective on the current understanding of the protein aggregation pathology in plectin‐related disorders and provides a basis for further pharmacological intervention studies addressing protein homeostasis.

## Conflicts of Interest

The authors declare no conflicts of interest.

## Supporting information


**Data S1** Counts.

**Data S2** DESeq2 Results.

**Data S3** GSEA and Genes.

**Data S4** GSEA and Autophagy.

**Data S5** GSEA and Genes2.

**Figure S1** AI‐based evaluation of SQSTM1 signal in human muscle samples and ultrastructural visualization of plectin‐deficient (MCK‐Cre/cKO) mouse muscles and wild‐type controls. (A) Artificial intelligence (AI)‐based evaluation of SQSTM1 signal intensities within individual myofibers using MIRA Vision. Whole muscle sections from human biopsies were scanned, myofibers automatically identified in an AI‐generated mask, and signal intensities obtained and binned (bin size = 0.1). Histograms represent the frequency distribution of binned intensities obtained from a control section (*n* = 4355 fibres) and EBS‐MD patient 1 (*n* = 579 fibres). Note the expanded distribution of the histogram and a shift towards the right (i.e., higher intensities) for the EBS‐MD patient muscle. (B) Representative lower magnification electron micrographs of soleus muscle cross sections obtained from 40‐week‐old MCK‐Cre/cKO mice. Note the occurrence of various degradative vacuoles (asterisks), including pathologically enlarged vacuoles that are partially filled with glycogen and/or membrane remnants (arrows). In addition, pathologically altered mitochondria with inclusions can be observed (arrowhead). Scale bars: 1 μm. (C) Representative electron micrographs of soleus muscle cross sections obtained from 40‐week‐old wild‐type mice. Note the tightly ordered appearance of myofibrils in wild‐type muscle. Scale bars: 500 nm.
**Figure S2**: Gene set enrichment analysis (GSEA) of hallmark and autophagy pathways. RNA‐Seq was performed on soleus muscles from wild‐type and MCK‐Cre/cKO mice. (A) Barplot showing differentially regulated biological processes and pathways of GSEA hallmark analysis. (B) Barplot showing differentially regulated pathways from supervised GSEA focusing on autophagy. For (A and B), adjusted p‐values (padj) < 0.05 were considered significant. Bars in blue indicate significant, bars in red non‐significant enrichment of gene sets. A positive normalized enrichment score (NES) value indicates enrichment in the MCK‐Cre/cKO group, a negative NES indicates enrichement in the wild‐type group.
**Figure S3**: Expression of TFEB, proteins required for the induction of autophagy and proteasomal subunits, and proteasomal activities in 13‐week‐old plectin‐deficient muscles. (A) Immunoblotting of muscle lysates from 13‐week‐old wild‐type and MCK‐Cre/cKO mice using antibodies to TFEB and GAPDH. Signal intensities of upper (corresponding to the phosphorylated versions, pTFEB) and lower (non‐phosphorylated, TFEB) protein bands were densitometrically measured and normalized to the total protein content as analysed by Coomassie staining (not shown). Mean ± SEM; *n* = 8. (B) Immunoblotting of wild‐type and MCK‐Cre/cKO muscle lysates using antibodies mTOR, ULK1, ATG7, Beclin‐1, ATG5, ATG3, and GAPDH. Signal intensities of protein bands were densitometrically measured and normalized to the total protein content as analysed by Coomassie staining (not shown). Mean ± SEM; n = 8–10. (C) RNA‐Seq analysis of mouse soleus muscle (as also shown in Figure 3A). Volcano plot illustrates differentially expressed genes in MCK‐Cre/cKO compared to wild‐type samples. All significantly up‐ and downregulated genes are highlighted in red; the dotted line represents the cut‐off with *p* = 0.01. Significantly up‐ and downregulated genes from the KEGG pathway “mmu03050 Proteasome” are highlighted in yellow and listed on the right. logFC, log fold change; *n* = 5 animals per genotype. (D) Immunoblotting of wild‐type and MCK‐Cre/cKO muscle lysates, derived from 13‐week‐old animals, using antibodies to 20S α1, 2, 3, 5, 6, and 7 proteasomal subunits (PSMA), and GAPDH. Signal intensities of protein bands were densitometrically measured and normalized to the total protein content as analysed by Coomassie staining (not shown). Mean ± SEM; *n* = 8. (E) Chymotrypsin‐, trypsin‐, and caspase‐like proteasomal activities as assessed in Figure 3D were normalized to the proteasomal protein content as analysed by immunoblotting (not shown). Mean ± SEM; samples were measured as triplicates, *n* = 3 animals each.
**Figure S4**: Evaluation of protein quality control mechanisms in muscles from aged mice. (A) Immunoblotting of muscle lysates from 40‐week‐old wild‐type and MCK‐Cre/cKO animals using antibodies mTOR, ULK1, ATG7, Beclin‐1, ATG5, ATG3, and GAPDH. Signal intensities of protein bands were densitometrically measured and normalized to the total protein content as analysed by Coomassie staining (not shown). Mean ± SEM; *n* = 6–8. (B) Immunoblotting of muscle lysates from 40‐week‐old wild‐type and MCK‐Cre/cKO animals using antibodies to ubiquitin and GAPDH. Signal intensities of immunoblots were densitometrically measured and normalized to the total protein content as analysed by Coomassie staining (not shown). Mean ± SEM; *n* = 8. (C) Chymotrypsin‐, trypsin‐, and caspase‐like proteasomal activities were measured in wild‐type and MCK‐Cre/cKO muscle lysates derived from 40‐week‐old mice. Mean ± SEM; samples were measured as triplicates, *n* = 3 animals each. (D) Immunoblotting of wild‐type and MCK‐Cre/cKO muscle lysates, derived from 40‐week‐old animals, using antibodies to 20S α1, 2, 3, 5, 6, and 7 proteasomal subunits (PSMA), and GAPDH. Signal intensities of protein bands were densitometrically measured and normalized to the total protein content as analysed by Coomassie staining (not shown). Mean ± SEM; *n* = 8. Chymotrypsin‐, trypsin‐, and caspase‐like proteasomal activities as assessed in (C) were normalized to the proteasomal protein content as analysed by immunoblotting (not shown). Mean ± SEM; samples were measured as triplicates, *n* = 3 animals each. (D) Immunoblotting of muscle lysates obtained from 40‐week‐old wild‐type and MCK‐Cre/cKO animals using antibodies to LAMP2, BAG3, total and phosphorylated forms of SQSTM1, and GAPDH. Signal intensities of protein bands were densitometrically measured and normalized to the total protein content as analysed by Coomassie staining (not shown). Mean ± SEM; *n* = 7–8. (E) Immunoblotting of muscle lysates obtained from 40‐week‐old wild‐type and MCK‐Cre/cKO animals using antibodies to LC3 and GAPDH. Signal intensities of upper (non‐lipidated, LC3‐I) and lower (lipidated, LC3‐II) protein bands were densitometrically measured and normalized to the total protein content (as analysed by Coomassie staining, not shown). From these values, the LC3‐II to LC3‐I ratios were calculated. Mean ± SEM; *n* = 8. For (A‐F): *p < 0.05, **p < 0.01, ***p < 0.001 (two‐tailed, unpaired *t*‐test with Welch’s correction); ns, not significant.
**Figure S5**: Accumulation of SQSTM1 and degradative vacuoles in plectin‐deficient myoblasts, but unaltered autophagy regulation. (A) Immunostaining of immortalized (*p53‍* ^*−/−*^) murine plectin‐positive (*Plec* ^*+/+*^) and plectin‐deficient (*Plec‍* ^*−/−*^) myoblasts using antibodies SQSMT1. Nuclei were visualized with DAPI. Scale bars: 20 μm. SQSTM1 signal intensities in *Plec* ^*+/+*^ and *Plec‍* ^*−/−*^ myoblasts were calculated by normalizing the raw integrated densities (RawIntDens) to cell areas. Each dot represents a single cell, the line represents the median (*Plec* ^*+/+*^, *n* = 41 cells; *Plec‍* ^*−/−*^, *n* = 40 cells); ***p < 0.001 (two‐tailed Mann–Whitney test). (B) Representative electron micrographs of *Plec‍* ^*−/−*^ myoblasts. Note the occurrence of degradative vacuoles (arrowhead) and membrane whirls (arrows), partially presenting with large vacuoles (asterisk). Scale bars: 250 nm. (C) Real‐time quantitative PCR (RT‐qPCR) analyses of ULK1 (*Ulk1)*, Beclin‐1 (*Becn1*), LAMP2 (*Lamp2*), BAG3 (*Bag3*), SQSTM1 (*Sqstm1*), LC3A (*Map 1lc3a*), and LC3B (*Map 1lc3b*) mRNA expression. Relative gene expression values are depicted as logFC and were normalized to *Tbp* and *Hprt.* Samples were measured as triplicates, *n* = 3 experiments. (D) Immunoblotting of *Plec* ^*+/+*^ and *Plec‍* ^*−/−*^ myoblast cell lysates using antibodies to mTOR, ULK1, Beclin‐1, and GAPDH. Signal intensities of protein bands were densitometrically measured and normalized to the total protein content as analysed by Coomassie staining (not shown). Mean ± SEM; *n* = 8. (E) Immunoblotting of *Plec* ^*+/+*^ and *Plec‍* ^*−/−*^ myoblast cell lysates using antibodies to ubiquitin and GAPDH. Signal intensities of protein bandswere densitometrically measured and normalized to the total protein content as analysed by Coomassie staining (not shown). Mean ± SEM; n = 8. For (D and E): *p < 0.05 (two‐tailed, unpaired *t*‐test with Welch’s correction); ns, not significant.
**Figure S6**: Evaluation of proteasomal activities and autophagic marker proteins in plectin‐deficient myoblasts. (A) Immortalized *Plec* ^*+/+*^ and *Plec‍* ^*−/−*^ myoblasts were either left untreated or treated with 50 μM MG132 for 4 or 8 h (h). Immunoblotting of cell lysates using antibodies to ubiquitin and GAPDH. Signal intensities of protein bands were densitometrically measured and normalized to the total protein content as analysed by Coomassie staining (not shown). Mean ± SEM; *n* = 4. (B) Chymotrypsin‐, trypsin‐, and caspase‐like proteasomal activities were measured in *Plec* ^*+/+*^ and *Plec‍* ^*−/−*^ myoblast cell lysates. Mean ± SEM; samples were measured as triplicates, *n* = 2 experiments. (C) Immunoblotting of *Plec* ^*+/+*^ and *Plec‍* ^*−/−*^ myoblast cell lysates using antibodies to 20S α1, 2, 3, 5, 6, and 7 proteasomal subunits (PSMA), and GAPDH. Signal intensities of protein bands were densitometrically measured and normalized to the total protein content as analysed by Coomassie staining (not shown). Mean ± SEM; *n* = 8. Chymotrypsin‐, trypsin‐, and caspase‐like proteasomal activities as assessed in (B) were normalized to the proteasomal protein content as analysed by immunoblotting (not shown). Mean ± SEM; samples were measured as triplicates, *n* = 3 animals each. (D) Immunoblotting of *Plec* ^*+/+*^ and *Plec‍* ^*−/−*^ myoblast cell lysates using antibodies to LAMP2, BAG3, total and phosphorylated forms of SQSTM1, and GAPDH. Signal intensities of protein bands were densitometrically measured and normalized to the total protein content as analysed by Coomassie staining (not shown). Mean ± SEM; *n* = 7–8. (E) Immunoblotting of *Plec* ^*+/+*^ and *Plec‍* ^*−/−*^ myoblast cell lysates using antibodies to LC3 and GAPDH. Signal intensities of upper (non‐lipidated, LC3‐I) and lower (lipidated, LC3‐II) protein bands were densitometrically measured and normalized to the total protein content (as analysed by Coomassie staining, not shown). From these values, the LC3‐II to LC3‐I ratios were calculated. Mean ± SEM; *n* = 8. For (A ‐E): *p < 0.05, **p < 0.01, ***p < 0.001 (two‐tailed, unpaired *t*‐test with Welch’s correction); ns, not significant.
**Figure S7**: Impaired autophagic flux in *Plec‍* ^*−/−*^ myoblast cell lines. (A) mCherry‐EGFP‐LC3B‐expressing *Plec* ^*+/+*^ and *Plec‍* ^*−/−*^ myoblasts were treated with 200 nM bafilomycin A1 (Baf A1) for 3 h. Scale bars: 20 μm. Red:green signal ratios of control (ctrl)‐ and Baf A1‐treated mCherry‐EGFP‐LC3B‐expressing *Plec* ^*+/+*^ and *Plec‍* ^*−/−*^ myoblasts. Box plots show the median and Tukey whiskers (*Plec* ^*+/+*^, *n* = 202/263 [control/Baf A1]; *Plec‍* ^*−/−*^, *n* = 142/202 [control/Baf A1] cells). (B) mCherry‐EGFP‐LC3B‐expressing *Plec* ^*+/+*^ and *Plec‍* ^*−/−*^ myoblasts were treated with 50 μM chloroquine (CQ) or 200 nM Baf A1 for 24 h. Note the massive swelling of vesicles in both *Plec* ^*+/+*^ and *Plec‍* ^*−/−*^ cells as well as the increased bright yellow signals compared to 3 h CQ‐treated cells shown in Figure 5B. Scale bars: 20 μm. Red:green signal ratios of ctrl‐ and 24 h CQ‐ and Baf A1‐treated mCherry‐EGFP‐LC3B‐expressing *Plec* ^*+/+*^ and *Plec‍* ^*−/−*^ myoblasts. Box plots show the median and Tukey whiskers (*Plec* ^*+/+*^, *n* = 113/149/177 [control/CQ/Baf A1]; *Plec‍* ^*−/−*^, *n* = 112/131/129 [control/CQ/Baf A1] cells). (C) mCherry‐EGFP‐LC3B‐expressing *Plec* ^*+/+*^ and *Plec‍* ^*−/−*^ myoblasts were treated with 9 mM 3‐methyladenine (3‐MA) for 24 h. Scale bars: 20 μm. Red:green signal ratios ctrl‐ and 24 h 3‐MA‐treated mCherry‐EGFP‐LC3B‐expressing *Plec* ^*+/+*^ and *Plec‍* ^*−/−*^ myoblasts. Box plots show the median and Tukey whiskers (*Plec* ^*+/+*^, *n* = 185/226 [control/3‐MA]; *Plec‍* ^*−/−*^, *n* = 176/196 [control/3‐MA] cells). (D) mCherry‐EGFP‐LC3B‐expressing *Plec* ^*+/+*^ and *Plec‍* ^*−/−*^ myoblasts were starved for 3 h. Scale bars: 20 μm. Red:green signal ratios of ctrl‐ and 3 h‐starved mCherry‐EGFP‐LC3B‐expressing *Plec* ^*+/+*^ and *Plec‍* ^*−/−*^ myoblasts: dotted lines represent the median values of the respective cells at control conditions. Box plots show the median and Tukey whiskers (*Plec* ^*+/+*^, *n* = 103/99 [control/starved]; *Plec‍* ^*−/−*^, *n* = 89/92 [control/starved] cells). For (A‐D): *p < 0.05, **p < 0.01, ***p < 0.001 (two‐way ANOVA of the ranked dataset with Tukey’s post hoc correction for multiple comparisons); ns, not significant.
**Figure S8**: Chloroquine treatment of wild‐type and MCK‐Cre/cKO mice. (A) Immunostaining using antibodies to SQSTM1 of frozen muscle sections prepared from wild‐type and MCK‐Cre/cKO mice treated for 4 h ante mortem with either 0.9% saline‐ (control) or 10 mg/mL CQ. Nuclei were visualized with DAPI. Panels on the right are magnifications of the boxed areas indicated in the panels on the left. Note enhanced SQSTM1 signals in wild‐type muscles upon CQ‐treatment, whereas the SQSTM1 signals in MCK‐Cre/cKO muscles remain similar to saline‐treated samples. Scale bars: 20 μm, magnifications 10 μm. (B) Immunoblotting of saline‐ (Ctrl) or CQ‐treated wild‐type and MCK‐Cre/cKO muscle lysates using antibodies to BAG3, total and phosphorylated forms of SQSTM1, LC3, and GAPDH. (C) Signal intensities of protein bands as shown in (B) were densitometrically measured and normalized to the total protein content as analysed by Coomassie staining (not shown). Mean ± SEM; *n* = 3–4; all *P* values are indicated (unpaired, two‐tailed *t*‐test with Welch’s correction). (D) Representative electron micrographs of soleus muscle cross sections obtained from saline‐ or CQ‐treated wild‐type and MCK‐Cre/cKO mice. Note the regular engulfment of cargo (arrow) in saline‐treated wild‐type muscle as well as the formation of membrane whirls (asterisks) and swollen, degradative vacuoles (arrowhead) in CQ‐treated wild‐type muscles. Also note that both saline‐ and CQ‐treated plectin‐deficient muscles of MCK‐Cre/cKO mice display a comparable occurrence of swollen, degradative vacuoles (arrowhead) and membrane whirls (asterisks); no aggravation of autophagolytic changes was observed upon CQ‐treatment. Scale bars: 500 nm. (E) Soleus muscle sections from saline‐ or CQ‐treated wild‐type and MCK‐Cre/cKO mice were stained with acid phosphatase (AP) and subjected to AI‐based evaluation of AP signal intensities within individual myofibers using MIRA Vision. Note the increased AP staining (red) within CQ‐treated wild‐type, as well as saline‐ and CQ‐treated MCK‐Cre/cKO myofibers. Scale bars: 25 μm. Wild‐type, *n* = 1129/1359 fibres [Ctrl/CQ]; MCK‐Cre/cKO, *n* = 852/1611 fibres [Ctrl/CQ]; two animals each; ***p < 0.001 (two‐way ANOVA of the ranked dataset with Tukey’s post hoc correction for multiple comparisons); ns, not significant.

**Data S6** Supplementary Methods.
