# Supplementary material for: Impaired Autophagic Flux in Skeletal Muscle of Plectin‐Related Epidermolysis Bullosa Simplex With Muscular Dystrophy
Source: J Cachexia Sarcopenia Muscle. 2025 Jul 10;16(4):e70001. doi: 10.1002/jcsm.70001 (PMC12246382; doi:10.1002/jcsm.70001)
Supplement: Supplementary file 6 — Figure S1 AI‐based evaluation of SQSTM1 signal in human muscle samples and ultrastructural visualization of plectin‐deficient (MCK‐Cre/cKO) mouse muscles and wild‐type controls. (A) Artificial intelligence (AI)‐based evaluation of SQSTM1 signal intensities within individual myofibers using MIRA Vision. Whole muscle sections from human biopsies were scanned, myofibers automatically identified in an AI‐generated mask, and signal intensities obtained and binned (bin size = 0.1). Histograms represent the frequency distribution of binned intensities obtained from a control section (n = 4355 fibres) and EBS‐MD patient 1 (n = 579 fibres). Note the expanded distribution of the histogram and a shift towards the right (i.e., higher intensities) for the EBS‐MD patient muscle. (B) Representative lower magnification electron micrographs of soleus muscle cross sections obtained from 40‐week‐old MCK‐Cre/cKO mice. Note the occurrence of various degradative vacuoles (asterisks), including pathologically enlarged vacuoles that are partially filled with glycogen and/or membrane remnants (arrows). In addition, pathologically altered mitochondria with inclusions can be observed (arrowhead). Scale bars: 1 μm. (C) Representative electron micrographs of soleus muscle cross sections obtained from 40‐week‐old wild‐type mice. Note the tightly ordered appearance of myofibrils in wild‐type muscle. Scale bars: 500 nm. Figure S2: Gene set enrichment analysis (GSEA) of hallmark and autophagy pathways. RNA‐Seq was performed on soleus muscles from wild‐type and MCK‐Cre/cKO mice. (A) Barplot showing differentially regulated biological processes and pathways of GSEA hallmark analysis. (B) Barplot showing differentially regulated pathways from supervised GSEA focusing on autophagy. For (A and B), adjusted p‐values (padj) < 0.05 were considered significant. Bars in blue indicate significant, bars in red non‐significant enrichment of gene sets. A positive normalized enrichment score (NES) value indicates [file JCSM-16-e70001-s005.docx]

**Impaired autophagic flux in skeletal muscle of plectin-related epidermolysis bullosa simplex with muscular dystrophy**

*Journal of Cachexia, Sarcopenia and Muscle*

Michaela M. Zrelski^a^, Margret Eckhard^a^, Petra Fichtinger^a^, Sabrina Hösele^a^, Andy Sombke^a^, Leonid Mill^b^, Monika Kustermann^c^, Wolfgang M. Schmidt^c^, Fiona Norwood^d^, Ursula Schlötzer-Schrehardt^e^, Gerhard Wiche^f^, Rolf Schröder^g^, and Lilli Winter^a^

^a^Division of Cell and Developmental Biology, Center for Anatomy and Cell Biology, Medical University of Vienna, Vienna, Austria

^b^MIRA Vision Microscopy GmbH, Wangen, Germany

^c^Neuromuscular Research Group, Division of Cell and Developmental Biology, Center for Anatomy and Cell Biology, Medical University of Vienna, Vienna, Austria

^d^ Department of Neurology, Ruskin Wing, King's College Hospital, London, UK

^e^Department of Ophthalmology, University Hospital Erlangen, Friedrich-Alexander University Erlangen-Nürnberg, Erlangen, Germany

^f^Department of Biochemistry and Cell Biology, Max Perutz Laboratories, University of Vienna,

Vienna, Austria

^g^Institute of Neuropathology, University Hospital Erlangen, Friedrich-Alexander University Erlangen-Nürnberg, Erlangen, Germany

Lilli Winter: phone: +43-1-4016037502, e-mail: [lilli.winter@meduniwien.ac.at](mailto:lilli.winter@meduniwien.ac.at)

**Supplemental Information**

**
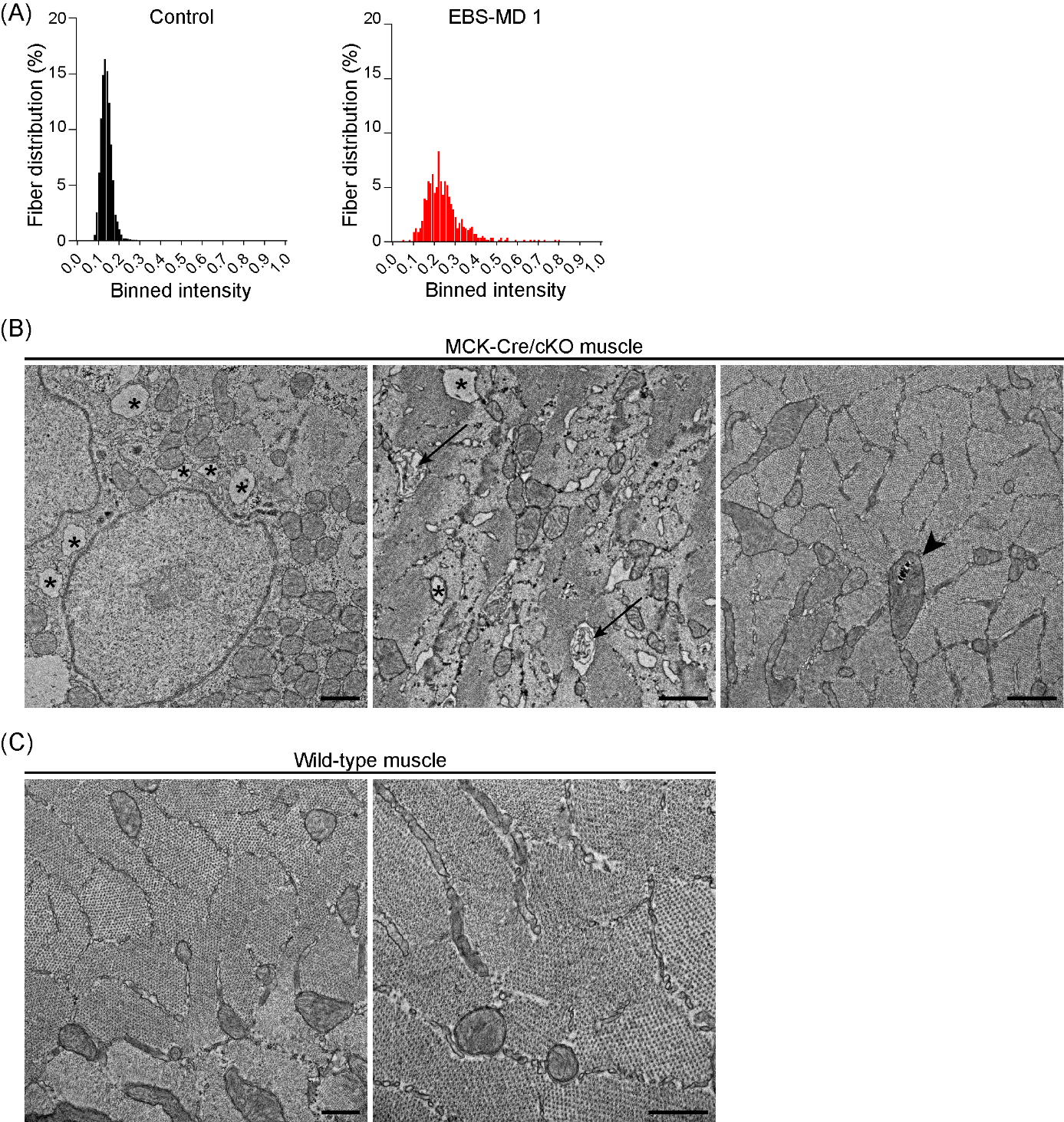
**

**Figure S1:** AI-based evaluation of SQSTM1 signal in human muscle samples and ultrastructural visualization of plectin-deficient (MCK-Cre/cKO) mouse muscles and wild-type controls. (A) ) Artificial intelligence (AI)-based evaluation of SQSTM1 signal intensities within individual myofibers using MIRA Vision. Whole muscle sections from human biopsies were scanned, myofibers automatically identified in an AI-generated mask, and signal intensities obtained and binned (bin size = 0.1). Histograms represent the frequency distribution of binned intensities obtained from a control section (n = 4355 fibers) and EBS-MD patient 1 (n = 579 fibers). Note the expanded distribution of the histogram and a shift towards the right (i.e. higher intensities) for the EBS-MD patient muscle. (B) Representative lower magnification electron micrographs of soleus muscle cross sections obtained from 40-week-old MCK-Cre/cKO mice. Note the occurrence of various degradative vacuoles (asterisks), including pathologically enlarged vacuoles that are partially filled with glycogen and/or membrane remnants (arrows). In addition, pathologically altered mitochondria with inclusions can be observed (arrowhead). Scale bars: 1 µm. (C) Representative electron micrographs of soleus muscle cross sections obtained from 40-week-old wild-type mice. Note the tightly ordered appearance of myofibrils in wild-type muscle. Scale bars: 500 nm.

**
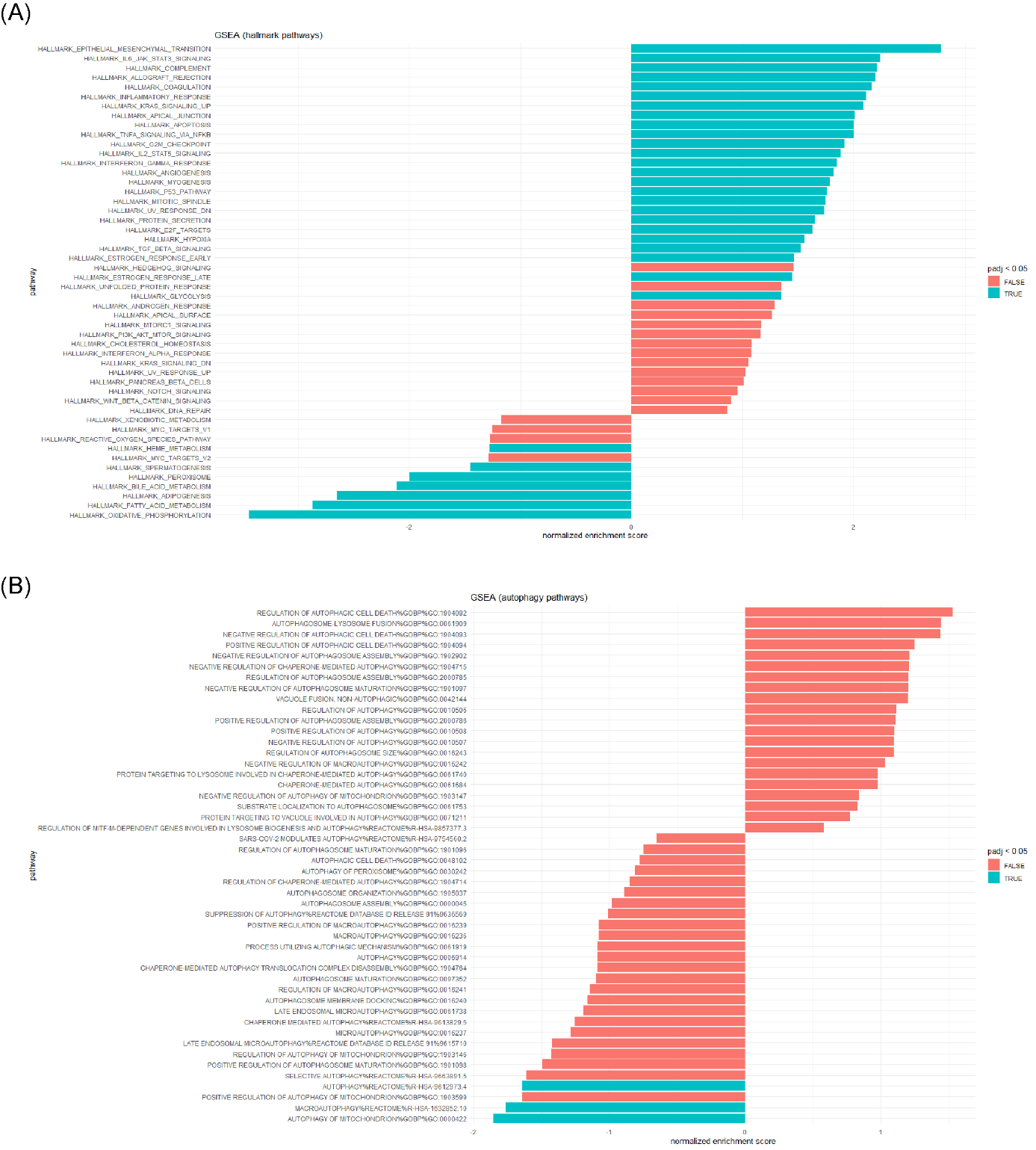
**

**Figure S2:** Gene set enrichment analysis (GSEA) of hallmark and autophagy pathways. RNA-Seq was performed on soleus muscles from wild-type and MCK-Cre/cKO mice. (A) Barplot showing differentially regulated biological processes and pathways of GSEA hallmark analysis. (B) Barplot showing differentially regulated pathways from supervised GSEA focusing on autophagy. For (A and B), adjusted p-values (padj) < 0.05 were considered significant. Bars in blue indicate significant, bars in red non-significant enrichment of gene sets. A positive normalized enrichment score (NES) value indicates enrichment in the MCK-Cre/cKO group, a negative NES indicates enrichement in the wild-type group.

**
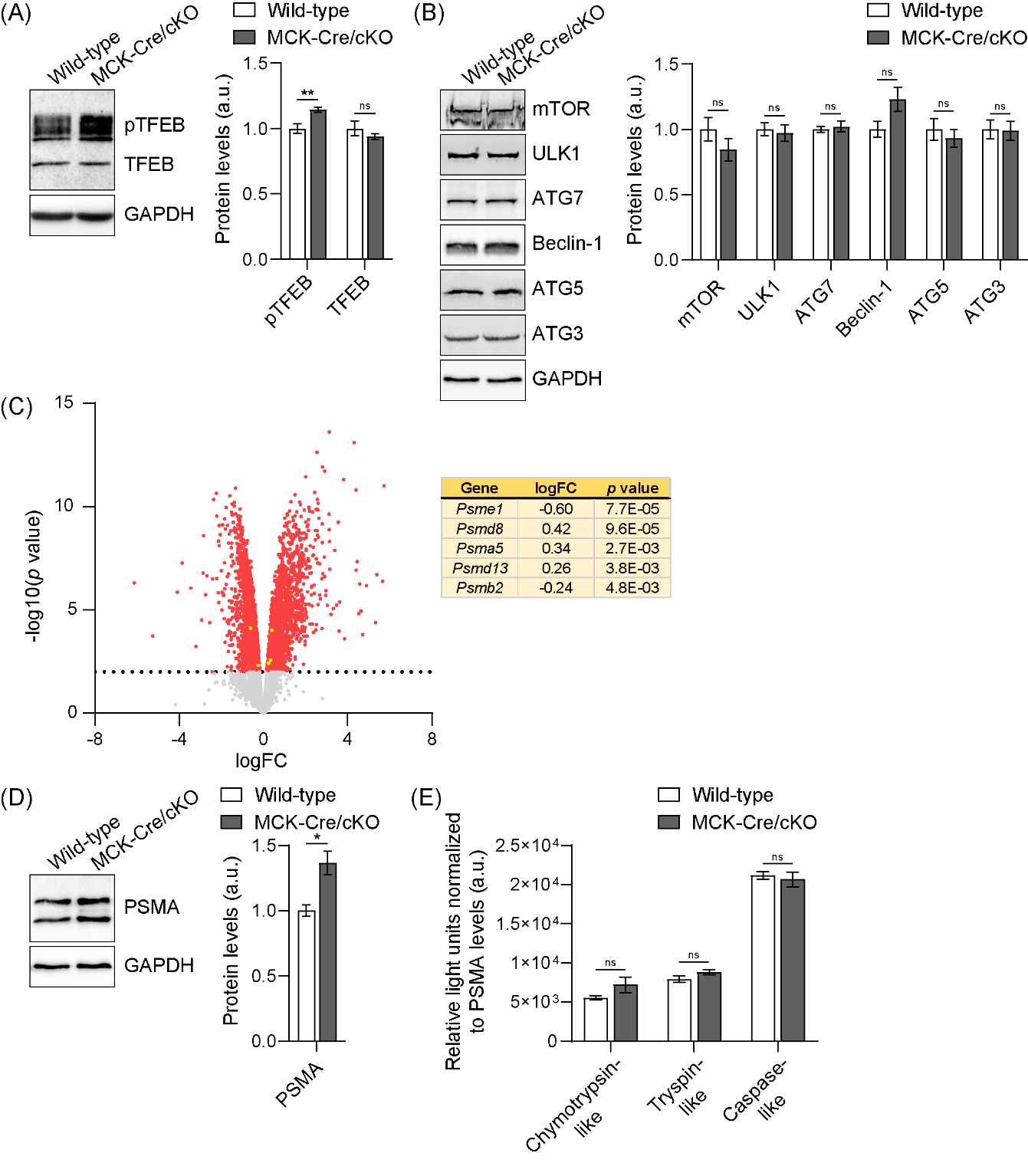
**

**Figure S3:** Expression of TFEB, proteins required for the induction of autophagy and proteasomal subunits, and proteasomal activities in 13-week-old plectin-deficient muscles. (A) Immunoblotting of muscle lysates from 13-week-old wild-type and MCK-Cre/cKO mice using antibodies to TFEB and GAPDH. Signal intensities of upper (corresponding to the phosphorylated versions, pTFEB) and lower (non-phosphorylated, TFEB) protein bands were densitometrically measured and normalized to the total protein content as analyzed by Coomassie staining (not shown). Mean ± SEM; n = 8. (B) Immunoblotting of wild-type and MCK-Cre/cKO muscle lysates using antibodies mTOR, ULK1, ATG7, Beclin-1, ATG5, ATG3, and GAPDH. Signal intensities of protein bands were densitometrically measured and normalized to the total protein content as analyzed by Coomassie staining (not shown). Mean ± SEM; n = 8-10. (C) RNA-Seq analysis of mouse soleus muscle (as also shown in Figure 3A). Volcano plot illustrates differentially expressed genes in MCK-Cre/cKO compared to wild-type samples. All significantly up- and downregulated genes are highlighted in red; the dotted line represents the cut-off with *P* = 0.01. Significantly up- and downregulated genes from the KEGG pathway “mmu03050 Proteasome” are highlighted in yellow and listed on the right. logFC, log fold change; n = 5 animals per genotype. (D) Immunoblotting of wild-type and MCK-Cre/cKO muscle lysates, derived from 13-week-old animals, using antibodies to 20S α1, 2, 3, 5, 6, and 7 proteasomal subunits (PSMA), and GAPDH. Signal intensities of protein bands were densitometrically measured and normalized to the total protein content as analyzed by Coomassie staining (not shown). Mean ± SEM; n = 8. (E) Chymotrypsin-, trypsin-, and caspase-like proteasomal activities as assessed in Figure 3D were normalized to the proteasomal protein content as analyzed by immunoblotting (not shown). Mean ± SEM; samples were measured as triplicates, n = 3 animals each.


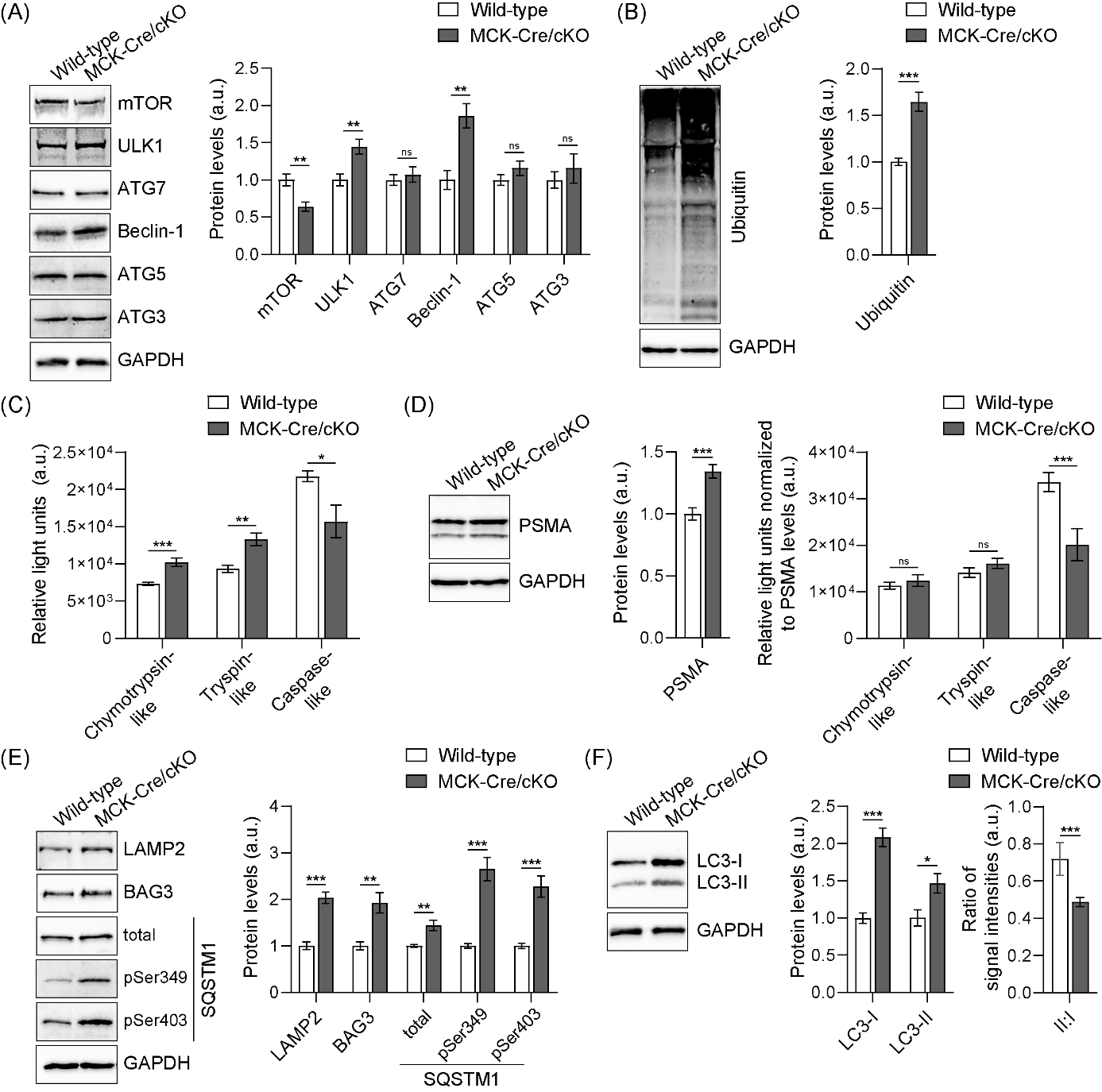


**Figure S4:** Evaluation of protein quality control mechanisms in muscles from aged mice. (A) Immunoblotting of muscle lysates from 40-week-old wild-type and MCK-Cre/cKO animals using antibodies mTOR, ULK1, ATG7, Beclin-1, ATG5, ATG3, and GAPDH. Signal intensities of protein bands were densitometrically measured and normalized to the total protein content as analyzed by Coomassie staining (not shown). Mean ± SEM; n = 6-8. (B) Immunoblotting of muscle lysates from 40-week-old wild-type and MCK-Cre/cKO animals using antibodies to ubiquitin and GAPDH. Signal intensities of immunoblots were densitometrically measured and normalized to the total protein content as analyzed by Coomassie staining (not shown). Mean ± SEM; n = 8. (C) Chymotrypsin-, trypsin‑, and caspase-like proteasomal activities were measured in wild-type and MCK-Cre/cKO muscle lysates derived from 40-week-old mice. Mean ± SEM; samples were measured as triplicates, n = 3 animals each. (D) Immunoblotting of wild-type and MCK-Cre/cKO muscle lysates, derived from 40-week-old animals, using antibodies to 20S α1, 2, 3, 5, 6, and 7 proteasomal subunits (PSMA), and GAPDH. Signal intensities of protein bands were densitometrically measured and normalized to the total protein content as analyzed by Coomassie staining (not shown). Mean ± SEM; n = 8. Chymotrypsin-, trypsin-, and caspase-like proteasomal activities as assessed in (C) were normalized to the proteasomal protein content as analyzed by immunoblotting (not shown). Mean ± SEM; samples were measured as triplicates, n = 3 animals each. (D) Immunoblotting of muscle lysates obtained from 40-week-old wild-type and MCK-Cre/cKO animals using antibodies to LAMP2, BAG3, total and phosphorylated forms of SQSTM1, and GAPDH. Signal intensities of protein bands were densitometrically measured and normalized to the total protein content as analyzed by Coomassie staining (not shown). Mean ± SEM; n = 7-8. (E) Immunoblotting of muscle lysates obtained from 40-week-old wild-type and MCK-Cre/cKO animals using antibodies to LC3 and GAPDH. Signal intensities of upper (non-lipidated, LC3-I) and lower (lipidated, LC3-II) protein bands were densitometrically measured and normalized to the total protein content (as analyzed by Coomassie staining, not shown). From these values, the LC3-II to LC3-I ratios were calculated. Mean ± SEM; n = 8. For (A-F): **P* < 0.05, ***P* < 0.01, ****P* < 0.001 (two-tailed, unpaired *t*-test with Welch’s correction); ns, not significant.


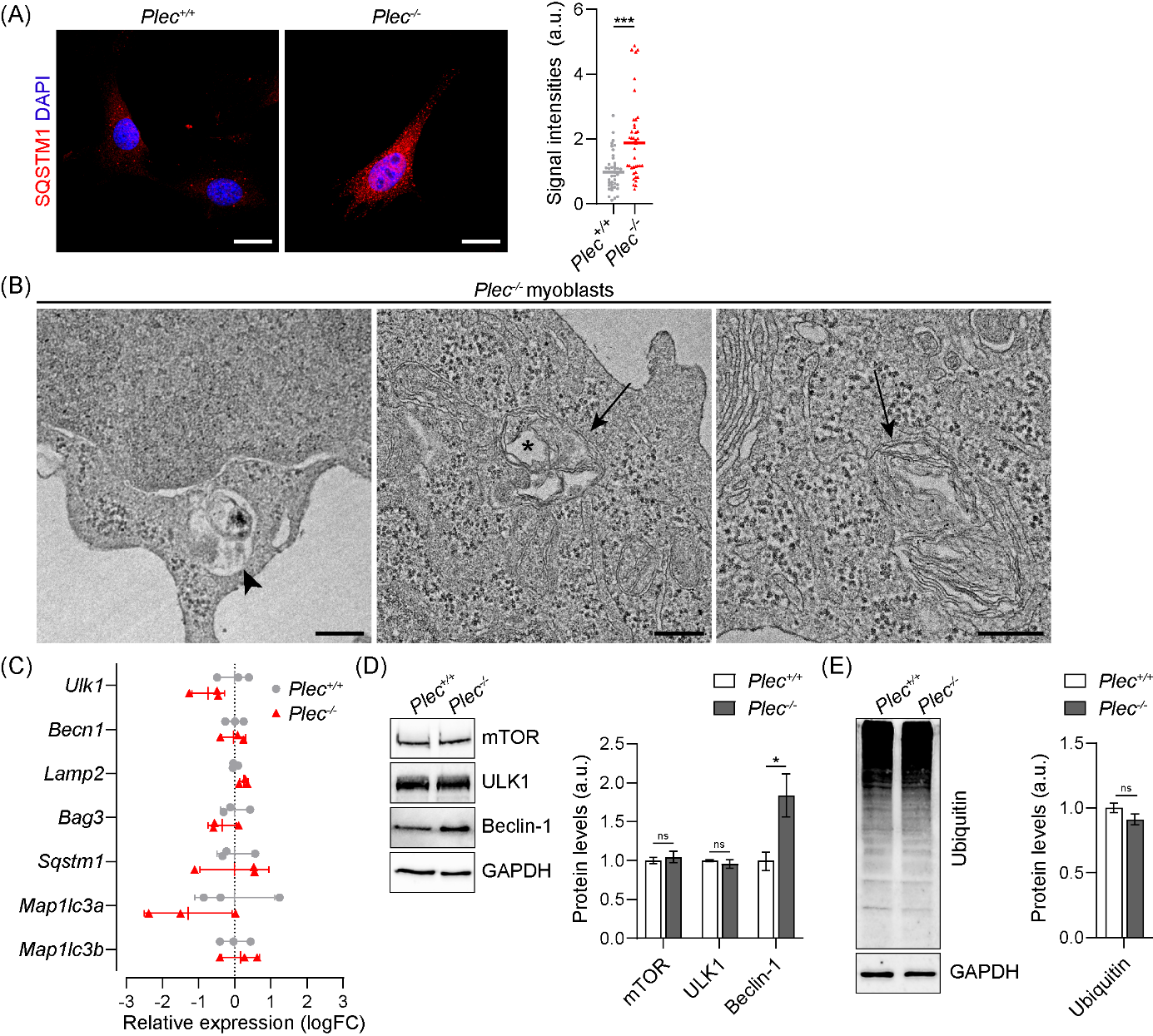


**Figure S5:** Accumulation of SQSTM1 and degradative vacuoles in plectin-deficient myoblasts, but unaltered autophagy regulation. (A) Immunostaining of immortalized (*p53‍^‑/‑^*) murine plectin-positive (*Plec^+/+^*) and plectin-deficient (*Plec‍^‑/‑^*) myoblasts using antibodies SQSMT1. Nuclei were visualized with DAPI. Scale bars: 20 µm. SQSTM1 signal intensities in *Plec^+/+^* and *Plec‍^‑/‑^* myoblasts were calculated by normalizing the raw integrated densities (RawIntDens) to cell areas. Each dot represents a single cell, the line represents the median (*Plec^+/+^*, n = 41 cells; *Plec‍^‑/‑^*, n= 40 cells); ****P* < 0.001 (two-tailed Mann-Whitney test). (B) Representative electron micrographs of *Plec‍^‑/‑^* myoblasts. Note the occurrence of degradative vacuoles (arrowhead) and membrane whirls (arrows), partially presenting with large vacuoles (asterisk). Scale bars: 250 nm. (C) Real-time quantitative PCR (RT-qPCR) analyses of ULK1 (*Ulk1)*, Beclin-1 (*Becn1*), LAMP2 (*Lamp2*), BAG3 (*Bag3*), SQSTM1 (*Sqstm1*), LC3A (*Map1lc3a*), and LC3B (*Map1lc3b*) mRNA expression. Relative gene expression values are depicted as logFC and were normalized to *Tbp* and *Hprt.* Samples were measured as triplicates, n = 3 experiments. (D) Immunoblotting of *Plec^+/+^* and *Plec‍^‑/‑^* myoblast cell lysates using antibodies to mTOR, ULK1, Beclin-1, and GAPDH. Signal intensities of protein bands were densitometrically measured and normalized to the total protein content as analyzed by Coomassie staining (not shown). Mean ± SEM; n = 8. (E) Immunoblotting of *Plec^+/+^* and *Plec‍^‑/‑^* myoblast cell lysates using antibodies to ubiquitin and GAPDH. Signal intensities of protein bandswere densitometrically measured and normalized to the total protein content as analyzed by Coomassie staining (not shown). Mean ± SEM; n = 8. For (D and E): **P* < 0.05 (two-tailed, unpaired *t*-test with Welch’s correction); ns, not significant.


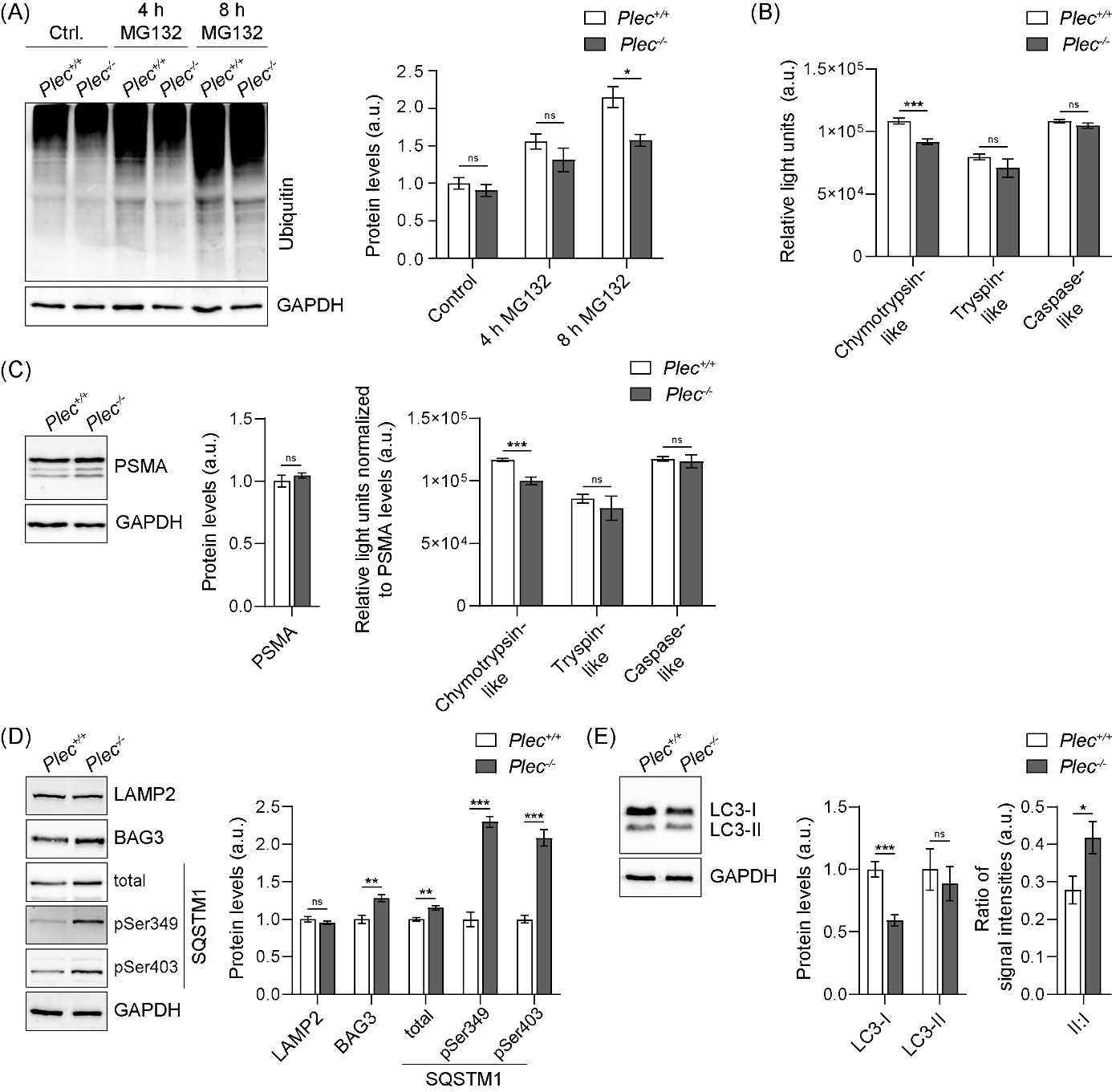


**Figure S6:** Evaluation of proteasomal activities and autophagic marker proteins in plectin-deficient myoblasts. (A) Immortalized *Plec^+/+^* and *Plec‍^‑/‑^* myoblasts were either left untreated or treated with 50 µM MG132 for 4 or 8 hours (h). Immunoblotting of cell lysates using antibodies to ubiquitin and GAPDH. Signal intensities of protein bands were densitometrically measured and normalized to the total protein content as analyzed by Coomassie staining (not shown). Mean ± SEM; n = 4. (B) Chymotrypsin-, trypsin-, and caspase-like proteasomal activities were measured in *Plec^+/+^* and *Plec‍^‑/‑^* myoblast cell lysates. Mean ± SEM; samples were measured as triplicates, n = 2 experiments. (C) Immunoblotting of *Plec^+/+^* and *Plec‍^‑/‑^* myoblast cell lysates using antibodies to 20S α1, 2, 3, 5, 6, and 7 proteasomal subunits (PSMA), and GAPDH. Signal intensities of protein bands were densitometrically measured and normalized to the total protein content as analyzed by Coomassie staining (not shown). Mean ± SEM; n = 8. Chymotrypsin-, trypsin-, and caspase-like proteasomal activities as assessed in (B) were normalized to the proteasomal protein content as analyzed by immunoblotting (not shown). Mean ± SEM; samples were measured as triplicates, n = 3 animals each. (D) Immunoblotting of *Plec^+/+^* and *Plec‍^‑/‑^* myoblast cell lysates using antibodies to LAMP2, BAG3, total and phosphorylated forms of SQSTM1, and GAPDH. Signal intensities of protein bands were densitometrically measured and normalized to the total protein content as analyzed by Coomassie staining (not shown). Mean ± SEM; n = 7-8. (E) Immunoblotting of *Plec^+/+^* and *Plec‍^‑/‑^* myoblast cell lysates using antibodies to LC3 and GAPDH. Signal intensities of upper (non-lipidated, LC3-I) and lower (lipidated, LC3-II) protein bands were densitometrically measured and normalized to the total protein content (as analyzed by Coomassie staining, not shown). From these values, the LC3-II to LC3-I ratios were calculated. Mean ± SEM; n = 8. For (A -**E**): **P* < 0.05, ***P* < 0.01, ****P* < 0.001 (two-tailed, unpaired *t*-test with Welch’s correction); ns, not significant.

**
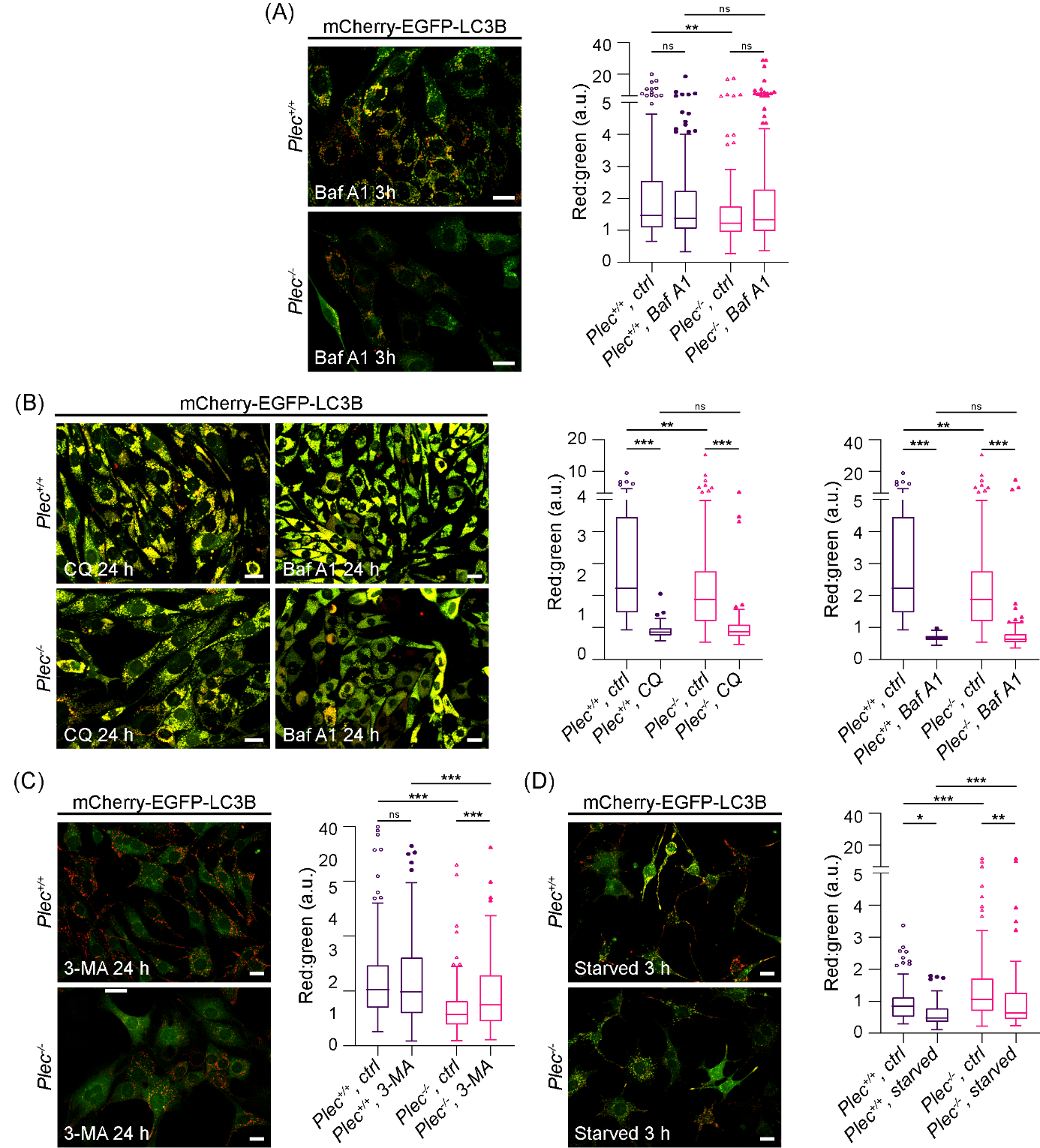
**

**Figure S7:** Impaired autophagic flux in *Plec‍^‑/‑^* myoblast cell lines. (A) mCherry-EGFP-LC3B-expressing *Plec^+/+^* and *Plec‍^‑/‑^* myoblasts were treated with 200 nM bafilomycin A1 (Baf A1) for 3 h. Scale bars: 20 µm. Red:green signal ratios of control (ctrl)- and Baf A1-treated mCherry-EGFP-LC3B-expressing *Plec^+/+^* and *Plec‍^‑/‑^* myoblasts. Box plots show the median and Tukey whiskers (*Plec^+/+^*, n = 202/263 [control/Baf A1]; *Plec‍^‑/‑^*, n = 142/202 [control/Baf A1] cells). (B) mCherry-EGFP-LC3B-expressing *Plec^+/+^* and *Plec‍^‑/‑^* myoblasts were treated with 50 µM chloroquine (CQ) or 200 nM Baf A1 for 24 h. Note the massive swelling of vesicles in both *Plec^+/+^* and *Plec‍^‑/‑^* cells as well as the increased bright yellow signals compared to 3 h CQ-treated cells shown in Figure 5B. Scale bars: 20 µm. Red:green signal ratios of ctrl- and 24 h CQ- and Baf A1-treated mCherry-EGFP-LC3B-expressing *Plec^+/+^* and *Plec‍^‑/‑^* myoblasts. Box plots show the median and Tukey whiskers (*Plec^+/+^*, n = 113/149/177 [control/CQ/Baf A1]; *Plec‍^‑/‑^*, n = 112/131/129 [control/CQ/Baf A1] cells). (C) mCherry-EGFP-LC3B-expressing *Plec^+/+^* and *Plec‍^‑/‑^* myoblasts were treated with 9 mM 3-methyladenine (3-MA) for 24 h. Scale bars: 20 µm. Red:green signal ratios ctrl- and 24 h 3-MA-treated mCherry-EGFP-LC3B-expressing *Plec^+/+^* and *Plec‍^‑/‑^* myoblasts. Box plots show the median and Tukey whiskers (*Plec^+/+^*, n = 185/226 [control/3-MA]; *Plec‍^‑/‑^*, n = 176/196 [control/3-MA] cells). (D) mCherry-EGFP-LC3B-expressing *Plec^+/+^* and *Plec‍^‑/‑^* myoblasts were starved for 3 h. Scale bars: 20 µm. Red:green signal ratios of ctrl- and 3h-starved mCherry-EGFP-LC3B-expressing *Plec^+/+^* and *Plec‍^‑/‑^* myoblasts: dotted lines represent the median values of the respective cells at control conditions. Box plots show the median and Tukey whiskers (*Plec^+/+^*, n = 103/99 [control/starved]; *Plec‍^‑/‑^*, n = 89/92 [control/starved] cells). For (A-D): **P* < 0.05, ***P* < 0.01, ****P* < 0.001 (two-way ANOVA of the ranked dataset with Tukey’s post-hoc correction for multiple comparisons); ns, not significant.

**
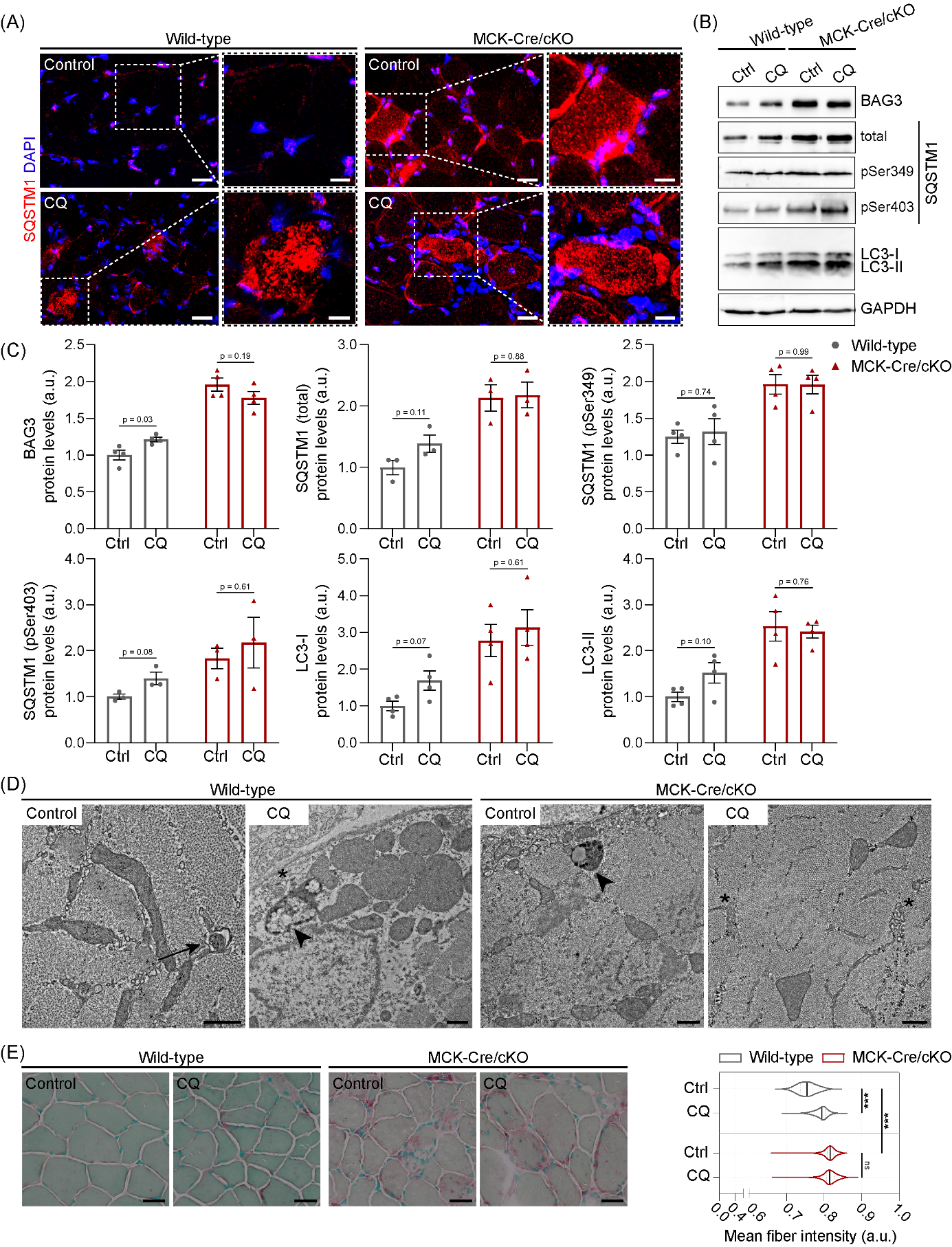
**

**Figure S8:** Chloroquine treatment of wild-type and MCK-Cre/cKO mice. (A) Immunostaining using antibodies to SQSTM1 of frozen muscle sections prepared from wild-type and MCK-Cre/cKO mice treated for 4 h ante mortem with either 0.9% saline- (control) or 10 mg/ml **CQ**. Nuclei were visualized with DAPI. Panels on the right are magnifications of the boxed areas indicated in the panels on the left. Note enhanced SQSTM1 signals in wild-type muscles upon CQ-treatment, whereas the SQSTM1 signals in MCK-Cre/cKO muscles remain similar to saline-treated samples. Scale bars: 20 µm, magnifications 10 µm. (B) Immunoblotting of saline- (Ctrl) or CQ-treated wild-type and MCK-Cre/cKO muscle lysates using antibodies to BAG3, total and phosphorylated forms of SQSTM1, LC3, and GAPDH. (C) Signal intensities of protein bands as shown in (B) were densitometrically measured and normalized to the total protein content as analyzed by Coomassie stainin (not shown). Mean ± SEM; n = 3-4; all *P* values are indicated (unpaired, two-tailed *t*-test with Welch’s correction). (D) Representative electron micrographs of soleus muscle cross sections obtained from saline- or CQ-treated wild-type and MCK-Cre/cKO mice. Note the regular engulfment of cargo (arrow) in saline-treated wild-type muscle as well as the formation of membrane whirls (asterisks) and swollen, degradative vacuoles (arrowhead) in CQ-treated wild-type muscles. Also note that both saline- and CQ-treated plectin-deficient muscles of MCK-Cre/cKO mice display a comparable occurrence of swollen, degradative vacuoles (arrowhead) and membrane whirls (asterisks); no aggravation of autophagolytic changes was observed upon CQ-treatment. Scale bars: 500 nm. (E) Soleus muscle sections from saline- or CQ-treated wild-type and MCK-Cre/cKO mice were stained with acid phosphatase (AP) and subjectd to AI-based evaluation of AP signal intensities within individual myofibers using MIRA Vision. Note the increased AP staining (red) within CQ-treated wild-type, as well as saline- and CQ-treated MCK-Cre/cKO myofibers. Scale bars: 25 µm. Wild-type, n = 1129/1359 fibers [Ctrl/CQ]; MCK-Cre/cKO, n = 852/1611 fibers [Ctrl/CQ]; two animals each; ****P* < 0.001 (two-way ANOVA of the ranked dataset with Tukey’s post-hoc correction for multiple comparisons); ns, not significant.
